# Pharmacological targeting of mitophagy via ALT001 improves herpes simplex virus 1 (HSV1)-mediated microglial inflammation and promotes amyloid β phagocytosis by restricting HSV1 infection

**DOI:** 10.7150/thno.105953

**Published:** 2025-03-31

**Authors:** Soo-Jin Oh, Young Yeon Kim, Ruiying Ma, Seok Tae Choi, Se Myeong Choi, Jong Hyun Cho, Ji-Yeun Hur, Yongjin Yoo, Kihoon Han, Hosun Park, Jeanho Yun, Ok Sarah Shin

**Affiliations:** 1 BK21 Graduate Program, Department of Biomedical Sciences, College of Medicine, Seoul, Republic of Korea.; 2 Department of Translational Biomedical Sciences, Graduate School of Dong-A University, Busan, Republic of Korea.; 3 Department of Neuroscience, Korea University College of Medicine, Seoul, Republic of Korea.; 4 Department of Microbiology, College of Medicine, Yeungnam University, Daegu, Republic of Korea.; 5 Department of Medicinal Biotechnology, College of Health Sciences, Dong-A University, Busan, Republic of Korea.; 6 Department of Biological Sciences, Ajou University, Suwon, Republic of Korea.; 7 Department of Convergence medicine, College of Medicine, Korea University Guro Hospital, Seoul, Republic of Korea.

**Keywords:** Microglia, herpes simplex virus 1 (HSV1), ALT001, alternative mitophagy, neurodegeneration

## Abstract

**Rationale:** One of the hallmarks of Alzheimer's disease (AD) is the accumulation of dysfunctional mitochondria. Herpes simplex virus type 1 (HSV1) may be a risk factor for the neuropathology linked to amyloid β (Aβ) accumulation. However, the mechanisms underlying HSV1-associated mitochondrial dysfunction in AD remain unclear. ALT001 is a novel drug that ameliorates AD-related cognitive impairment via ULK1/Rab9-mediated alternative mitophagy. In this study, we investigated the effects of ALT001 on the neurodegeneration-related microglial signatures associated with HSV1 infection.

**Methods:** Molecular mechanisms and physiological functions of mitophagy was investigated in HSV1-infected microglia, including primary murine and human embryonic stem cell (ESC)-derived microglia (ES-MG), as well as in a microglia-neuron co-culture system. Microglial gene signatures following HSV1 infection in the presence or absence of ALT001 were analyzed using bulk RNA sequencing, and the effects of ALT001 on microglial phagocytosis and microglia-mediated immune responses were further evaluated by flow cytometry and cytokine profiles.

**Results:** HSV1 infection inhibited PINK1/Parkin-mediated mitophagy via HSV1-encoded protein kinase US3, resulting in mitochondrial dysfunction in both human and mouse microglia. Furthermore, transcriptomic analysis of HSV1-infected microglia revealed an upregulation of distinct microglial genes associated with disease-associated microglia (DAM)-like phenotype and pro-inflammatory activity. Pharmacological targeting of mitophagy using ALT001 prevents mitochondrial damage caused by HSV1 through ULK1/Rab9-mediated pathway. Furthermore, ALT001-induced ULK1/Rab9-dependent mitophagy restricts HSV1 infection by activating interferon-mediated antiviral immunity. Consequently, ALT001 reduces HSV1-triggered neuroinflammation, recovers HSV1-altered microglial phagocytosis for Aβ, and efficiently reverses morphological and molecular abnormalities in HSV1-infected microglia by triggering mitophagy in ES-MG. ALT001 also suppressed HSV1-mediated Aβ accumulation and neurodegeneration in the microglia-neuron co-culture and cerebral organoid model.

**Conclusions:** In this study, we identified a critical molecular link between HSV1 and AD-related microglial dysfunction. Furthermore, our findings provide an evidence that therapeutic targeting of alternative mitophagy via ALT001 effectively interfere with HSV1-induced microglial dysfunction and alleviate neurodegeneration.

## Introduction

Microglia are resident macrophages in the brain that perform multiple functions supporting the central nervous system (CNS), such as regulating synaptic pruning and blood-brain barrier (BBB) permeability 1-3. Additionally, microglia safeguard neuronal function via microglia-neuron crosstalk 4. In healthy individuals, microglia are capable of effectively phagocytizing neurotoxic protein aggregates like amyloid β (Aβ) plaques and neurofibrillary tangles (NFTs). However, in patients with neurodegenerative diseases, particularly Alzheimer's disease (AD), microglia-mediated inflammatory cytokine production is increased and their phagocytic activity is impaired, leading to the accumulation of Aβ plaques and hyperphosphorylated tau in the brain 5-7. Furthermore, recent transcriptomic studies have highlighted the significance of a unique microglial subtype, namely disease-associated microglia (DAM), in the progression of AD 8. Given that microglia participate in the progression of neurodegenerative diseases via multiple mechanisms, it is important to explore microglia-targeted interventions and effective treatments for AD and other neurodegenerative disorders.

Herpes simplex virus type 1 (HSV1) is a neurotropic virus responsible for a wide spectrum of clinical complications ranging from harmless skin manifestations to herpes simplex encephalitis (HSE), which is the leading cause of viral encephalitis 9. HSV1 is usually acquired during childhood and causes lifelong infections because of its ability to establish latent infections in the trigeminal ganglion 10. Interestingly, it was anticipated that frequent reactivation of HSV1 in the brain would contribute to neurodegenerative phenotypes through elevated neuroinflammation 10, 11. For example, >70% of late-onset AD cases have latent HSV1 infection, and HSV1 infection increased the probability of early onset AD due to *apolipoprotein 4* (*APOE4*) genetic mutations 12-14. Additionally, DNA fragments of HSV1 were detected within Aβ plaque and HSV1 glycoprotein B showed highly homogeneous amino acid sequences similar to Aβ 15-17. Considering its close association with Aβ pathology, further studies are needed to understand HSV1-microglia interactions.

A hallmark of neurodegenerative diseases is the accumulation of dysfunctional mitochondria associated with microglia-induced inflammation. Evidence suggests a role for microglial mitophagy in controlling AD-associated behavioral and pathological hallmarks 18. Mitophagy contributes to mitochondrial quality control by removing dysfunctional mitochondria using the core autophagic machinery 19. In particular, PTEN-induced kinase 1 (PINK1)/Parkin-mediated mitophagy is ubiquitin-dependent and involves endoplasmic reticulum-derived autophagosome membranes following mitochondrial membrane depolarization. In contrast to PINK1/Parkin-mediated canonical mitophagy, Unc-51-like kinase 1 (ULK1)/Rab9-dependent alternative mitophagy is thought to be ubiquitin-independent and employs trans Golgi-derived autophagosome membranes independently of mitochondrial depolarization. Recently, a novel drug ALT001 was developed and shown to improve cognitive impairment in mouse models of AD by inducing mitochondrial biogenesis and stimulating ULK1/Rab9-dependent mitophagy 20. Given that mitophagy modulates various immune responses, viruses have developed multiple strategies to subvert mitophagy to facilitate replication. However, the mechanism through which HSV1 affects microglial mitophagy remains unclear.

To determine whether HSV1 manipulates mitochondrial homeostasis in microglia, we used multiparametric and high-throughput methods to quantify multifunctional roles of microglia in primary murine and human embryonic stem cell-derived microglia (ES-MG), as well as in a microglia-neuron co-culture system. We further focused on the role of the mitophagy-inducing drug, ALT001, during HSV1 infection of microglia. Our findings identify ALT001 as an anti-herpetic drug and a potential therapeutic agent against neurodegeneration caused by HSV1 infection.

## Results

### HSV1 inhibits microglial mitophagy via US3 resulting in mitochondrial dysfunction

**Figure 1:**
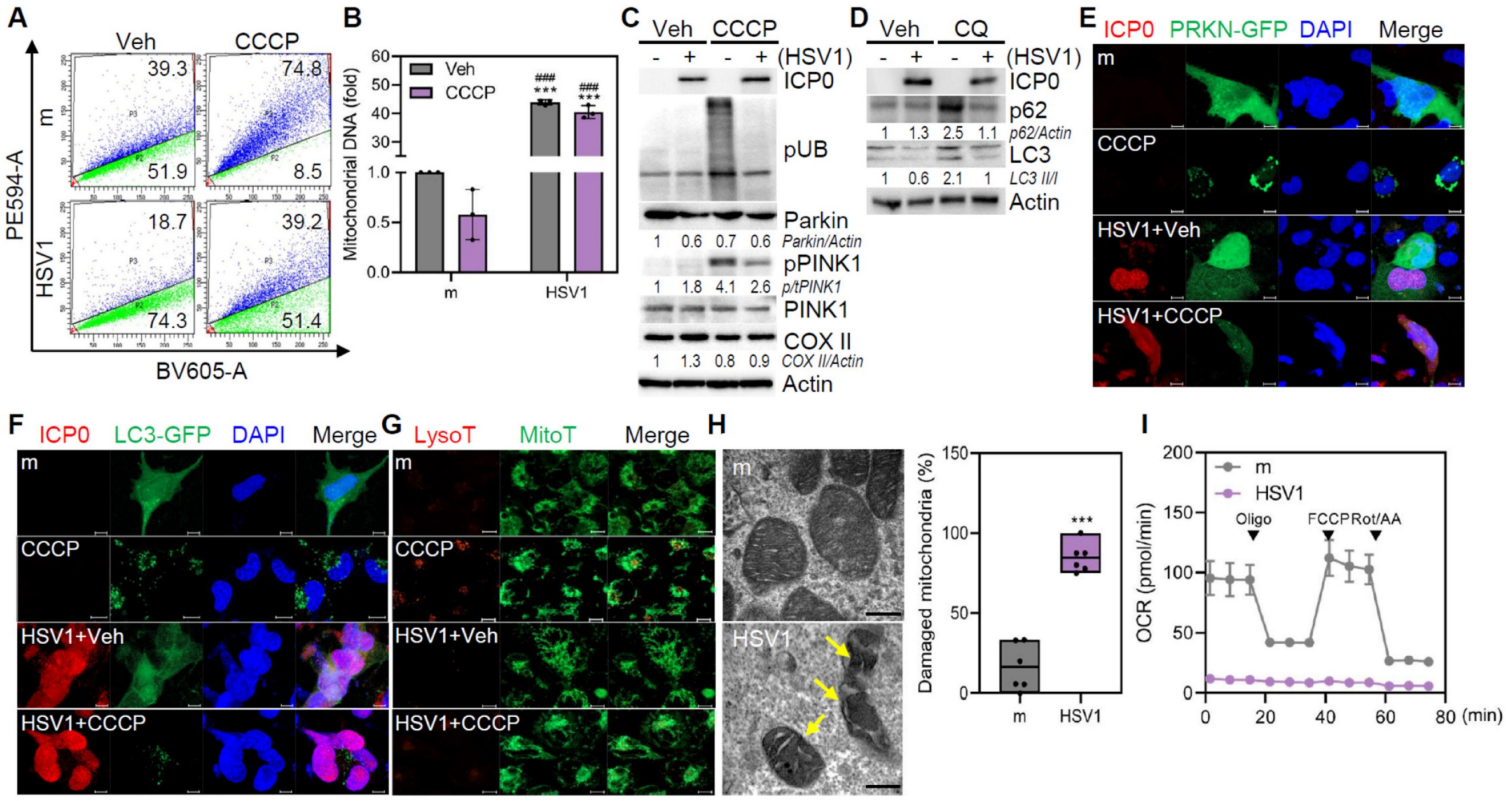
** HSV1 suppresses mitophagy in human microglia. A, B** Human microglial cells HMC3 stably expressing mitochondria-targeted Keima (HMC3-mt-Keima) were infected with HSV1 (MOI 1) for 24 h followed by 20 μM CCCP treatment. Following 2 h period, mitophagy was quantified by flow cytometry (A) and mitochondrial DNA was quantified using genomic DNA, and normalized against 18s ribosomal RNA (18s RNA) (B). **C** Proteins were extracted from HSV1-infected HMC3 at MOI 1 for 24 h followed by 20 μM CCCP treatment for 2 h. Representative immunoblots of HSV1 ICP0, phospho-ubiquitin (pUB; Ser56), Parkin, phospho-PINK1 (pPINK1), PINK1, mitochondria-encoded cytochrome c oxidase II (COX II), and Actin are shown. Semi-quantification of protein expression by densitometry is shown below the blot. **D** HMC3 were infected with HSV1 (MOI 1) for 24 h followed by 10 μM chloroquine (CQ) treatment for 8 h. Immunoblot analysis was performed to examine the levels of HSV1 ICP0, p62, LC3, and Actin.** E, F** HMC3 were transfected with Parkin (PRKN)-GFP (E) or LC3-GFP (F), followed by HSV1 (MOI 1) infection. At 24 hpi, cells were treated with 20 μM CCCP for 2 h. Representative immunofluorescence images showing Parkin (green), LC3 (green) and HSV1 ICP0 (red). Scale bar=10 μm. **G** Representative immunofluorescence images showing MitoTracker (green) and LysoTracker (red) staining. Scale bar=10 μm. **H** HMC3 were infected with HSV1 (MOI 1) for 48 h for transmission electron microscopy analysis The number of damaged mitochondria/total mitochondria was quantified. Scale bar=200 nm. **I** HMC3 were infected with HSV1 (MOI 1) for 48 h and mitochondrial respiration was determined using Cell Mito Stress test kit and analyzed with Seahorse XFp analyzer. OCR=oxygen consumption rate. All data represent the means ± SD of at least three independent experiments. Statistical analysis: one-way ANOVA with Dunnett's post-hoc correction (**B**) or Student's t-test (**H**). ***p < 0.001, versus mock (m)-infected and vehicle (Veh)-treated group. ^###^p < 0.001, versus mock (m)-infected and CCCP-treated group.

Multiple studies have demonstrated that HSV1 encodes several autophagy inhibitory proteins 21-23, but it is not yet known how HSV1 controls mitophagy, particularly in microglia. Therefore, we generated a human microglial HMC3 cell line expressing pH-dependent mitochondria-targeted Keima protein (mt-Keima; HMC3-mt-Keima), which was utilized to quantify mitophagy activity via analysis of fluorescence signals at acidic and neutral pH 24. HMC3 exhibited mitophagy activity of approximately 39.3% under basal conditions, whereas HSV1-infected HMC3 displayed a markedly reduced mitophagy activity of 18.7% (**Figure 1A**). Administration of carbonyl cyanide m-chlorophenyl hydrazine (CCCP) increased mitophagy in HMC3 by approximately 74.8%; however, HSV1-infected HMC3 showed a minimal increase in mitophagy in response to CCCP treatment. As a consequence of mitophagy inhibition by HSV1, mitochondrial DNA was upregulated 40-fold in HSV1-infected HMC3 compared to that in the mock control (**Figure 1B**), despite CCCP treatment. Similar to HMC3, murine immortalized microglia BV2 also showed increased mitochondrial DNA (mtDNA) content during HSV1 infection, regardless of CCCP treatment (**Figure S1A**-**B**).

HSV1-infected microglia displayed decreased Parkin ubiquitination and PINK1 phosphorylation (**Figure 1C, Figure S1C**). Furthermore, we observed impaired autophagic flux in HSV1-infected microglia, as evidenced by the reduced accumulation of LC3 II in chloroquine (CQ)-treated cells (**Figure 1D, Figure S1D**). Similar to Parkin and autophagy flux inhibition, HSV1 significantly reduced the number of Parkin and LC3 puncta caused by CCCP (**Figure 1E-F**). Additionally, HSV1 inhibited mitolysosome formation, as indicated by the reduced co-localization of LysoTracker and MitoTracker (**Figure 1G**). In HSV1-infected primary mouse microglia (pMG), the expression of mitophagy-related genes, such as *Pink1*, *Parkin*, *Bnip3*, and *Bnip3l* (encoding NIX), was inhibited, whereas the expression of mitochondrial proteins belonging to the *Tomm* family was generally increased (**Figure S1E**).

Next, we determined structural and functional changes in mitochondria following HSV1 infection. Transmission electron microscopy (TEM) indicated the presence of highly damaged mitochondria in response to HSV1 (**Figure 1H**). In correlation with the structural changes, oxygen consumption rate (OCR) was significantly downregulated by HSV1, indicating HSV1-driven dysfunction of microglial mitochondrial metabolism (**Figure 1I**). Taken together, these results indicate that HSV1 infection causes mitochondrial damage and dysfunction by interfering with PINK1/Parkin-dependent mitophagy.

**Figure 2:**
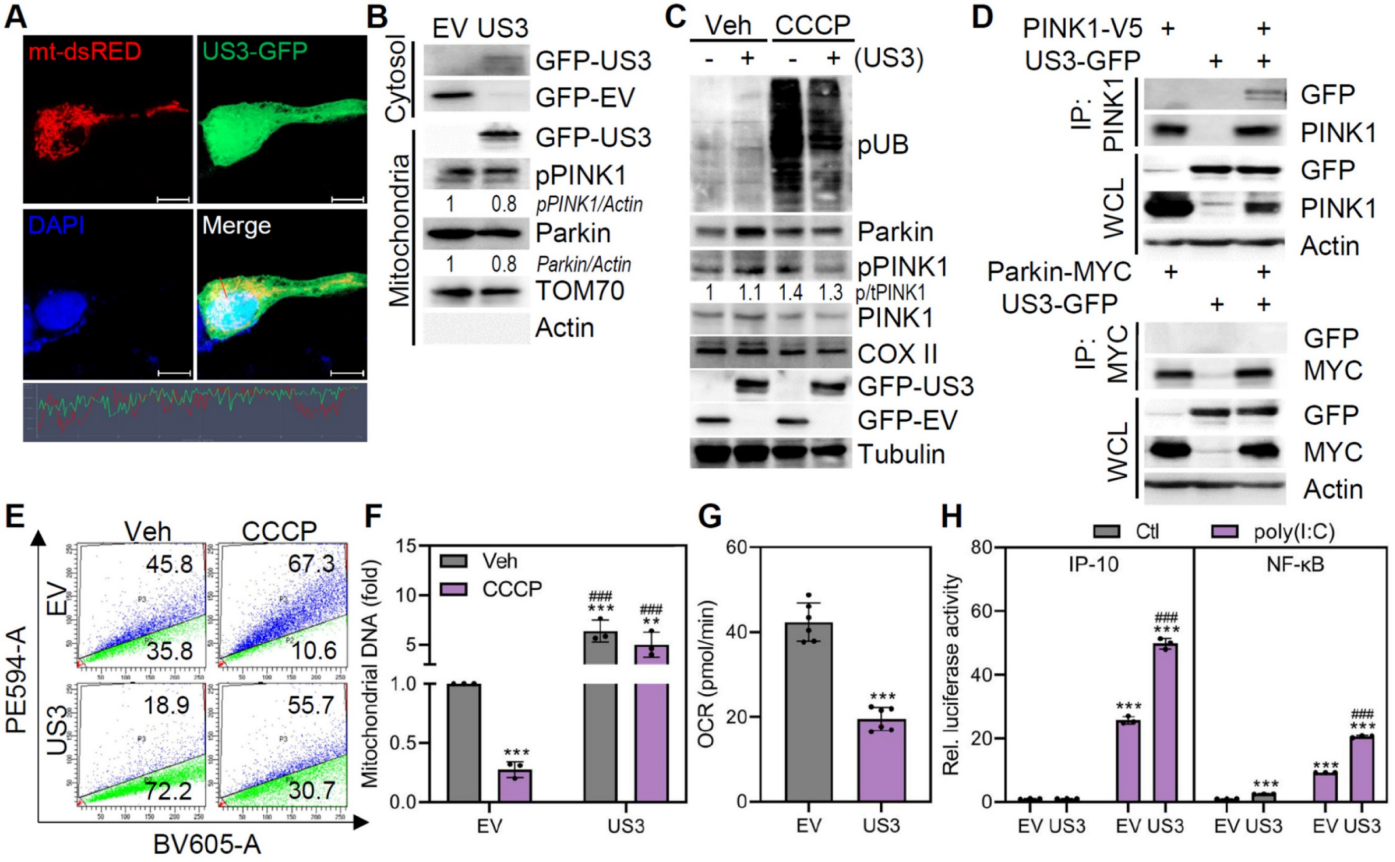
** HSV1-encoded US3 inhibits PINK1/Parkin-mediated mitophagy. A** Representative confocal images of cells expressing both mt-dsRED (red) and US3-GFP (green). Scale bar=10 μm. **B** Cytosol and mitochondria were fractionated from cells transfected with empty vector (EV)-GFP or US3-GFP and analyzed by immunoblotting to determine the relative levels of GFP-tagged US3 or EV, phospho-PINK1 (pPINK1), Parkin, TOM70, and Actin. Semi-quantification of protein expression by densitometry is indicated below the blot. **C** HMC3 transfected with EV-GFP or US3-GFP were treated with 10 μM CCCP for 2 h and immunoblot analysis was performed to examine the levels of phospho-ubiquitination (pUB; Ser56), Parkin, pPINK1, PINK1, COX II, GFP-tagged US3 or EV, and Tubulin. **D** HEK293T were co-transfected with PINK1-V5 or Parkin-MYC along with US3-GFP. Cell lysates were immunoprecipitated using antibodies against PINK1 and MYC-conjugated magnetic beads. **E** HMC3-mt-Keima transfected with pBHA-US3 plasmid were treated with 20 μM CCCP for 2 h. Mitophagic cells were quantified by flow cytometry. **F** HMC3 transfected with EV-GFP or US3-GFP were treated with 20 μM CCCP for 2 h and mitochondrial DNA levels were examined using genomic DNA, normalizing against 18s rRNA. **G** Mitochondrial respiration profiles were determined in cells transfected with EV-GFP or US3-GFP using XFp analyzer. OCR=oxygen consumption rate. **H** HMC3 were transfected with IP-10 and NF-κB reporter plasmids along with EV-GFP or US3-GFP. Cells were treated with 10 μg/ml Poly(I:C) for 6 h and the results of the luciferase reporter assay was shown. All data represent the means ± SD of at least three independent experiments. Statistical analysis: one-way ANOVA with Dunnett's post-hoc correction. **p < 0.01; ***p < 0.001, versus vehicle (Veh)-treated EV group. ^###^p < 0.001, versus CCCP or poly I:C-treated EV group.

To identify HSV1 proteins that contribute to mitophagy inhibition, we focused on the HSV1-encoded protein kinase US3, which is known to inhibit autophagy and localize to the mitochondria 23, 25. We confirmed the presence of HSV1 US3 in mitochondrial fractions (**Figure 2A-B**). In light of the finding that HSV1 impairs mitophagy, HSV1 US3 physically interacted with PINK1 but not with Parkin and prevented CCCP-induced Parkin ubiquitination and PINK1 phosphorylation (**Figure 2C-D**).

In HMC3-mt-Keima cells, HSV1 US3 overexpression significantly attenuated mitophagy under both basal and CCCP-treated conditions, and further increased mtDNA levels (**Figure 2E-F**). These data are consistent with the results obtained from HSV1-infected microglia. Given that other HSV1 proteins such as US11 was reported to suppress microglial mitophagy 26, we examined mitophagy-inhibitory effects of US11 using our experimental system. As shown by **Figure S2**, US11 inhibited mitophagy and we observed synergistic effects of US3 and US11 in inhibiting mitophagy. Furthermore, US3 overexpression triggered mitochondrial dysfunction as demonstrated by decreased OCR as a result of mitophagy suppression and enhanced poly(I:C)-stimulated inflammation as demonstrated by IP-10 and NF-κB promoter activities (**Figure 2G-H**). These findings demonstrate that US3 encoded by HSV1 limits mitophagy in microglia during HSV1 infection and that its interaction with PINK1 has the potential to inhibit mitophagy.

### HSV1-infected microglia undergo distinct transcriptional changes increasing microglial inflammation

**Figure 3:**
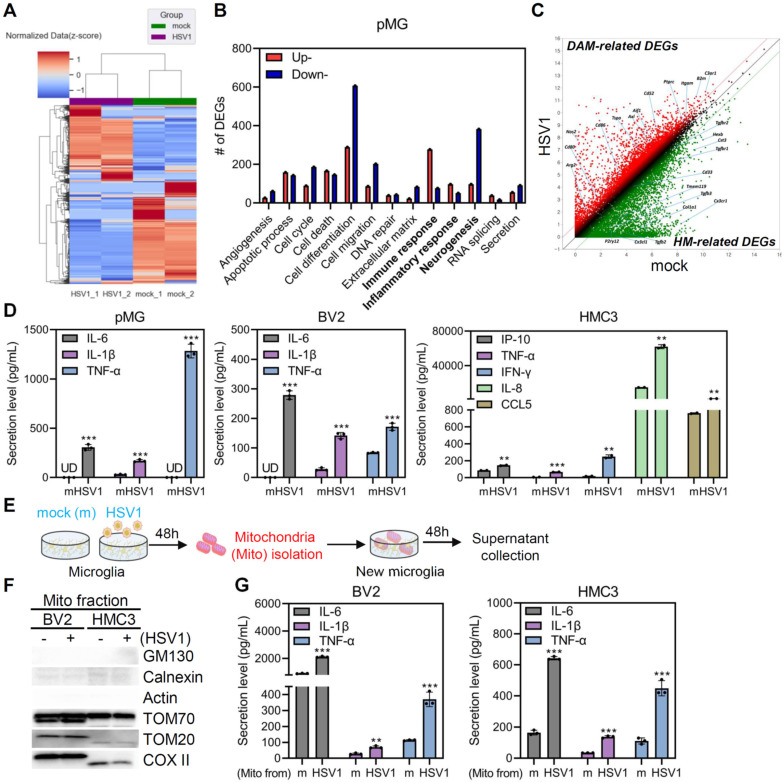
** Damaged mitochondria from HSV1-infected microglia induces inflammation. A** Primary murine microglia (pMG) were isolated, cultured and infected with mock (m) or HSV1 (MOI 10) for 48 h and bulk RNA sequencing was performed. A hierarchical clustering heatmap represents overall transcriptome signatures. **B** The number of differentially expressed genes (DEGs) according to GO term analysis; 2-fold upregulated or downregulated DEGs of HSV1-infected pMG compared to m-infected groups (*p* <0.05). **C** Scatter plot illustrating 2-fold upregulated (red dot) or downregulated (green dot) DEGs (*p* <0.05). HM=homeostatic microglia, DAM=disease-associated microglia. **D** pMG were infected with HSV1 (MOI 10) for 72 h, while BV2 or HMC3 were infected with HSV1 (MOI 1) for 48 h. Secretion levels of pro-inflammatory cytokines/chemokines including interleukin (IL)-6, IL-1β, and tumor necrosis factor (TNF)-α by pMG and BV2 were measured by ELISA. Interferon (IFN)-γ-induced protein 10 (IP-10), TNF-α, IFN-γ, IL-8 and Chemokine (C-C motif) ligand 5 (CCL5) secretion by HMC3 were measured by Luminex. **E** Experimental scheme showing that mitochondria fractionated from the m- or HSV1-infected cells were added to fresh microglial culture. **F** Representative immunoblot analysis confirming protein levels of GM130, Calnexin, Actin, TOM70, TOM20, and COX II. **G** BV2 or HMC3 were treated with mitochondria (Mito) from m- or HSV1-infected cells for 48 h. Secretion levels of IL-6, IL-1β, and TNF-α were examined by ELISA. All data represent the means ± SD of at least three independent experiments. Statistical analysis: Student's t-test. ^**^p < 0.01; ^***^p < 0.001, versus m-infected group

Emerging evidence suggests that microglia display plasticity and diversity with phenotypic heterogeneity 3. pMG were isolated from mouse pups (**Figure S3A**), and quantitative RT-PCR demonstrated that HSV1 successfully established the infection in pMG (**Figure S3B**). Bulk mRNA sequencing of HSV1-infected pMG was performed to characterize the microglial transcriptional landscape. Clustering of whole gene sets revealed that HSV1-infected cells displayed significantly different gene expression patterns than mock-infected cells (**Figure 3A**). Among differentially expressed genes (DEGs), 1,953 genes were downregulated and 1,658 genes were upregulated following HSV1 infection. Gene ontology (GO) and Kyoto Encyclopedia of Genes and Genomes (KEGG) pathway analyses revealed that HSV1 infection led to the upregulation of DEGs involved in immunological response and inflammatory pathways, while many neurogenesis-related DEGs were downregulated by HSV1 (**Figure 3B, Figure S4**). Microglia are broadly classified as homeostatic microglia (HM) and DAM 3, 5. HSV1-infected microglia exhibited a >2 fold decrease in 30 HM-related genes, including *P2ry13*, *P2ry12*, and *Cx3cr1*, but a >2 fold increase in 40 DAM-related genes, including *Axl*, *Aif1* (encoding IBA1), *B2m*, and *Nos2* (**Figure 3C**). Correlating with these, HSV1-infected BV2 and pMG produce higher secretions of pro-inflammatory cytokines and chemokines, including interleukin (IL)-6, IL-1β, and tumor necrosis factor (TNF)-α, while HSV1 infection led to elevated levels of interferon (IFN)-γ -induced protein 10 (IP-10), TNF-α, IFN-γ, IL-8 and Chemokine (C-C motif) ligand 5 (CCL5) in HMC3 (**Figure 3D**). These findings suggest that HSV1 infection alters microglial signatures of microglial homeostasis and innate immunity.

Next, we investigated whether the accumulation of damaged mitochondria caused by HSV1 infection leads to microglial phenotypic changes and inflammation 27, 28. Therefore, mitochondria were isolated from mock- or HSV1-infected microglia and added to fresh microglial cultures (**Figure 3E**). First, we confirmed the purity of the mitochondrial fraction by immunoblot analysis. Interestingly, mitochondria purified from HSV1-infected cells triggered significantly higher levels of IL-6, IL-1β, and TNF-α in fresh microglia cultures (**Figure 3F-G**). Taken together, HSV1-driven inhibition of mitophagy and the consequent accumulation of damaged mitochondria may activate inflammation in microglia, indicating the significance of mitophagy in modulating microglial phenotype.

### ALT001 prevents HSV1-associated mitochondrial damage via ULK1/Rab9-mediated mitophagy

**Figure 4:**
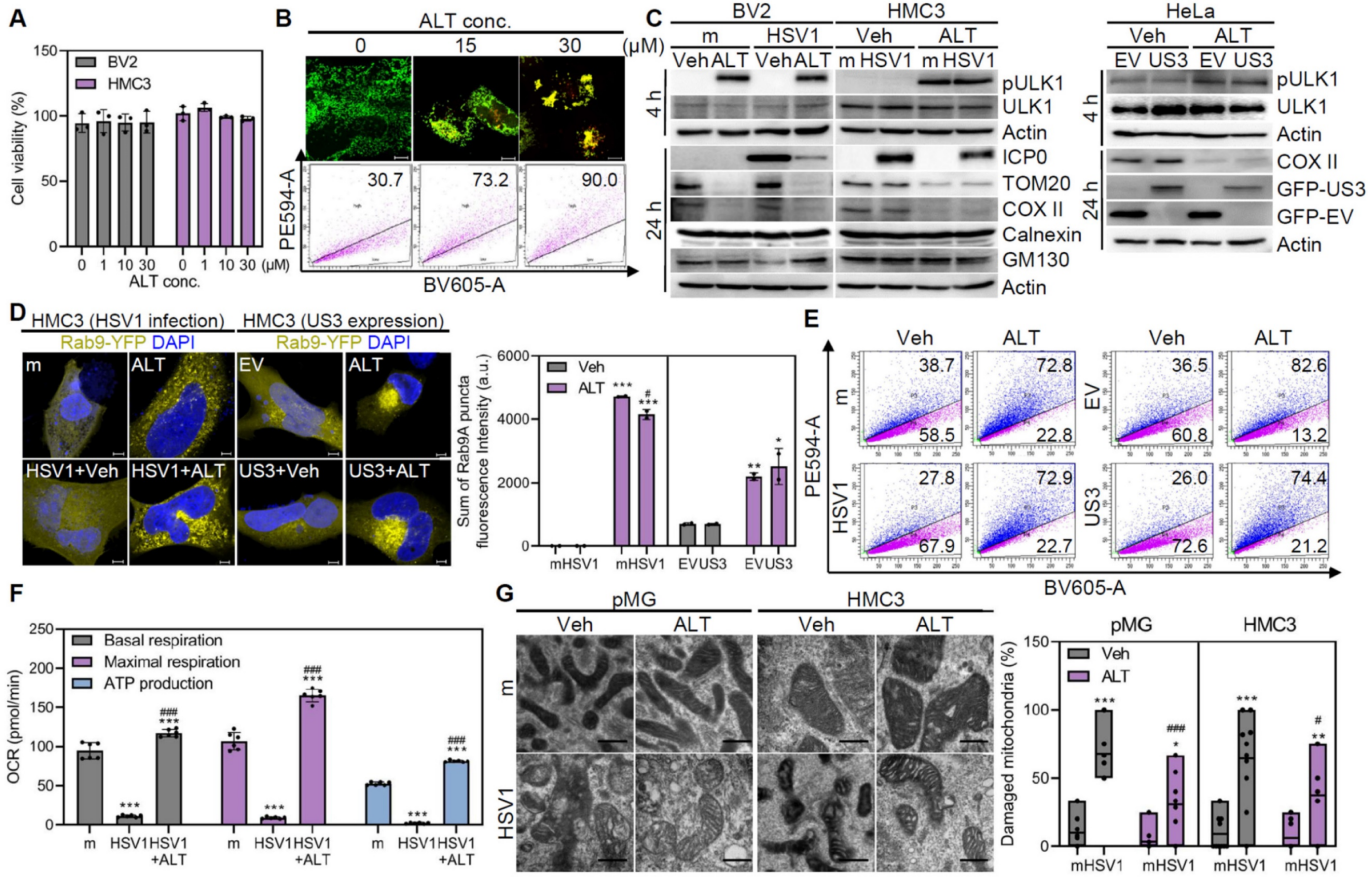
** ALT001 prevents HSV1-induced mitochondrial damage via ULK1/Rab9-mediated mitophagy. A** Cell viability assay was performed in ALT001 (ALT)-treated cells for 48 h. **B** HMC3-mt-Keima were treated with 0, 15, and 30 μM ALT for 24 h and mitophagic cells were examined by both flow cytometry and confocal microscopy. Scale bar=10 μm. **C** HSV1 (MOI 1) was inoculated in HMC3 or BV2 for 1 h and treated with 30 μM ALT. HeLa were transfected with EV-GFP or US3-GFP for 24 h and treated with 30 μM ALT. Representative immunoblot analysis was shown to assess the levels of phospho-ULK1 (pULK1; Ser 555), ULK1, HSV1 ICP0, TOM20, COX II, Calnexin, GM130, GFP-tagged US3 or EV, and Actin. **D** Rab9-YFP-expressing HMC3 were infected with HSV1 (MOI 1) or transfected with pBHA-US3. After infection or transfection, cells were treated with 30 μM ALT for 48 h and Rab9 puncta formation was measured by confocal microscopy. Fluorescence intensity was quantified using Zen Desk. Scale bar=5 μm. **E** HMC3-mt-Keima were infected with HSV1 at MOI 1 for 24 h or transfected with pBHA-US3 followed by 30 μM ALT. Mitophagic cells were examined by flow cytometry. **F** HSV1 was inoculated in HMC3 at MOI 1 for 1 h and cells were treated with 30 μM ALT. At 48 h, oxygen consumption rate (OCR) was measured by XFp analyzer. **G** Transmission electron microscopy analysis shows the quantification of damaged mitochondria/total mitochondria. Scale bar=200 nm. All data represent the means ± SD of at least three independent experiments. Statistical analysis: one-way ANOVA with Dunnett's post-hoc correction. *p < 0.05; **p < 0.01; ***p < 0.001, versus mock (m)-infected group. ^#^p < 0.05;^ ###^p < 0.001, versus HSV1-infected group.

We have previously reported that a novel mitophagy inducer, ALT001, specifically activates ULK1/Rab9-dependent alternative mitophagy in neurons, and alleviates neurodegenerative phenotypes in a mouse AD model 20. Here we tested whether ALT001 treatment activates ULK1/Rab9-mediated mitophagy in microglia. Similar to neuronal cells, ALT001 increased mitophagy activity in microglia in a dose-dependent manner, without cytotoxicity (**Figure 4A-B**). Furthermore, ALT001 treatment triggered the phosphorylation of ULK1 at Ser555 and the formation of Rab9 puncta, even in the presence of HSV1 infection or US3 expression, indicating that ALT001 markedly alleviated HSV1-mediated impairment of mitophagy (**Figure 4C**-**D**). In consequence of ULK1 and Rab9 activation, flow cytometry analysis of ALT001-activated mitophagy in HMC3-mt-Keima demonstrated that ALT001 successfully activated microglial mitophagy in HSV1-infected or US3-expressing cells (**Figure 4E**).

Next, the impact of ALT001 on structural or functional modifications in the mitochondria was investigated. Remarkably, ALT001 reversed the metabolic alterations caused by HSV1 infection and significantly decreased the number of damaged mitochondria (**Figure 4F**-**G**). In conclusion, ALT001 treatment prevents HSV1-induced mitochondrial damage and dysfunction in microglia by promoting ULK1/Rab9-mediated mitophagy.

### ALT001 provides an antiviral effect against HSV1

**Figure 5:**
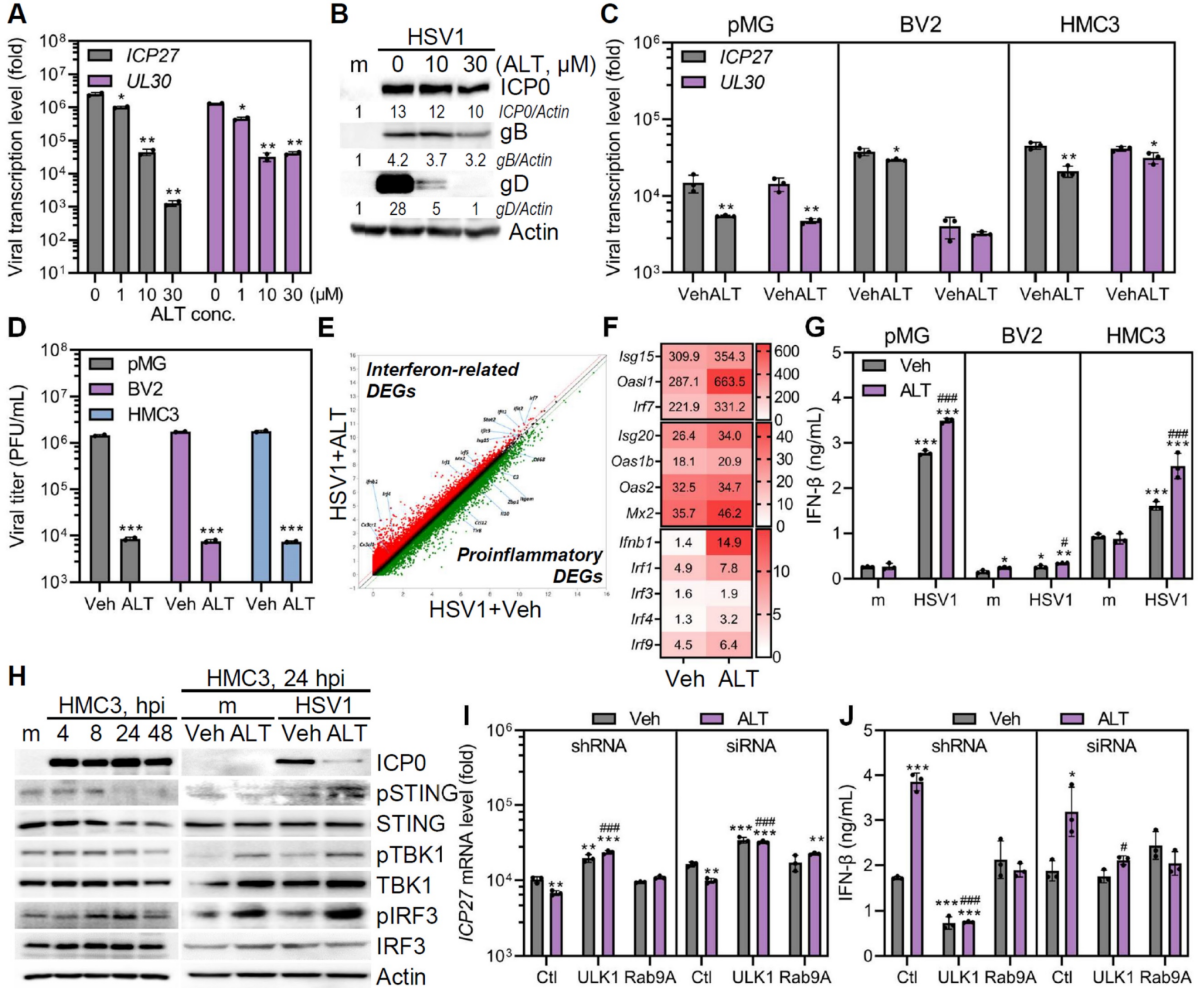
** ALT001-driven ULK1/Rab9-mediated mitophagy promotes antiviral function against HSV1 by triggering IFN response. A** Quantitative RT-PCR was performed to measure *HSV1 ICP27* and *UL30* transcripts in HSV1 (MOI 1)-infected Vero cells treated with various concentrations of ALT001 (ALT) for 48 h. **B** HSV-1 ICP0, gB, gD, and Actin levels were quantified by densitometry of immunoblots. **C, D** HSV1 was inoculated into primary microglia (pMG; MOI 10), BV2 (MOI 1), and HMC3 (MOI 1), and cells were treated with 30 μM ALT. *HSV1 ICP27* and *UL30* mRNA levels were analyzed by quantitative RT-PCR (C) and infectious viral loads were quantified by plaque assay (D). Statistical analysis: Student's t-test, *p < 0.05; **p < 0.01; ***p < 0.001, versus Vehicle (Veh)-treated group. **E** A scatter plot indicates ALT treatment in HSV1-infected pMG led to alteration of mRNA expression profiles including upregulation of interferon-signaling-related genes (red dots) and downregulation of pro-inflammatory genes (green dots). **F** A heatmap represents the expression levels of genes involved in antiviral responses. **G** The secretion level of IFN-β was measured by ELISA. Statistical analysis: one-way ANOVA with Dunnett's post-hoc correction. *p < 0.05; **p < 0.01; ***p < 0.001, versus Veh-treated mock (m)-infected group. ^#^p < 0.05; ^###^p < 0.001, versus Veh-treated HSV1-infected group. **H** Representative immunoblot analysis of HSV1 ICP0, phospho-STING (pSTING), STING, phospho-TBK1 (pTBK1), TBK1, phospho-IRF3 (pIRF3), IRF3, and Actin. **I, J** ULK1 or Rab9 knockdown in HMC3 was introduced by shRNAs or siRNAs and cells were infected with HSV1 in the presence or absence of 30 μM ALT. At 48 hpi, quantitative RT-PCR was performed to examine the transcript levels of *HSV ICP27* (I) and ELISA was performed to examine the secretion levels of IFN-β (J). All data represent the means ± SD of at least three independent experiments. Statistical analysis: one-way ANOVA with Dunnett's post-hoc correction. *p < 0.05; **p < 0.01; ***p < 0.001, versus control shRNA (Ctl)-transfected Veh-treated group. ^#^p < 0.05; ^###^p < 0.001, Ctl-transfected ALT-treated group.

Growing evidence suggests that viruses are capable of manipulating mitophagy to evade antiviral innate immunity 29-33. Therefore, we investigated the potential of ALT001 as an antiviral drug. Vero cells were exposed to HSV1, followed by ALT001 treatment to assess the antiviral activity of ALT001 against HSV1. Interestingly, ALT001 treatment significantly attenuated gene and protein expression of HSV1 in a dose-dependent manner (**Figure 5A**-**B**). Consistent with the observations in Vero cells, ALT001 administration to both human and mouse microglia significantly inhibited HSV1 gene expression and infectious viral propagation (**Figure 5C-D**). In our *in vitro* model, ALT001 exhibited potent antiviral effects against HSV1, considering that inhibitory concentration for 50% of viral replication (IC50) values of ALT001 was observed to be 1.33 μM, compared to acyclovir (ACV) with an IC50 of 15.64 μM (data not shown).

Next, ALT001-mediated activation of antiviral immunity was investigated by transcriptome analysis. HSV1-infected pMG with ALT001 administration had higher expression of genes involved in antiviral immunity, such as *Isg15*, *Oasl1*, *Irf7*, *Isg20*, *Mx2,* and *ifnb1*, than HSV-infected pMG alone (**Figure 5E**-**F**). In correlation with gene expression profiles, ALT001 significantly increased the secretion levels of IFN-β in microglia (**Figure 5G**). Considering that cyclic GMP-AMP synthase/stimulator of interferon genes (cGAS/STING) pathway is essential for early host defense against HSV1 and multiple HSV-1 proteins disrupt cGAS/STING pathway 34-36, we investigated whether ALT001 promoted activation of cGAS/STING pathway by immunoblot analysis. HSV1 infection led to rapid suppression of phospho-STING and subsequent reduction of phospho-TANK-binding kinase 1 (TBK1) at 8 hours post infection (hpi). (**Figure 5H**). Interestingly, ALT001 treatment led to an increase in the phosphorylation of STING, TBK1, and interferon regulatory factor 3 (IRF3) up to 24 hpi. This suggests that ALT001 triggered cGAS/STING-mediated interferon response to restrict HSV1 replication.

To explore whether ULK1/Rab9-mediated mitophagy contributes to the antiviral role of ALT001 during HSV1 infection, HMC3 were transfected with shRNA, siRNA-specific ULK1, or Rab9 (**Figure S5A**), followed by HSV1 infection with or without ALT001 treatment. As shown in **Figure 5I-J**, genetic depletion of Rab9 or ULK1 failed to induce the antiviral effect of ALT001 on HSV1, suggesting the importance of ULK1/Rab9 for ALT001 mode of action in limiting HSV1 replication. To confirm this result, we used the ULK1-specific inhibitor SBI-0206925 to validate the role of ULK1 in HSV1 infection (**Figure S5B**). We confirmed that SBI-0206925 specifically inhibited the phosphorylation of ULK1 at Ser555 and led to upregulation of HSV1 replication and titer even in the presence of ALT001 treatment, suggesting that ULK1 has a modulatory role in ALT001-mediated antiviral activities (**Figure S5B**-**E**).

HSV1 causes apoptotic cell death in microglia 37. Therefore, we investigated whether ALT001 affected HSV1-associated cell death. As expected, immunoblotting and flow cytometry analyses revealed that HSV1 infection led to significantly higher apoptotic cell death. However, ALT001 administration inhibited the apoptosis of HSV1-infected microglia (**Figure S6**). Collectively, ALT001 has antiviral effects against HSV1 and the ULK1/Rab9-mediated pathway is important for its antiviral role.

### ALT001 triggers transcriptional transition of microglia and alleviates microglial inflammation

**Figure 6:**
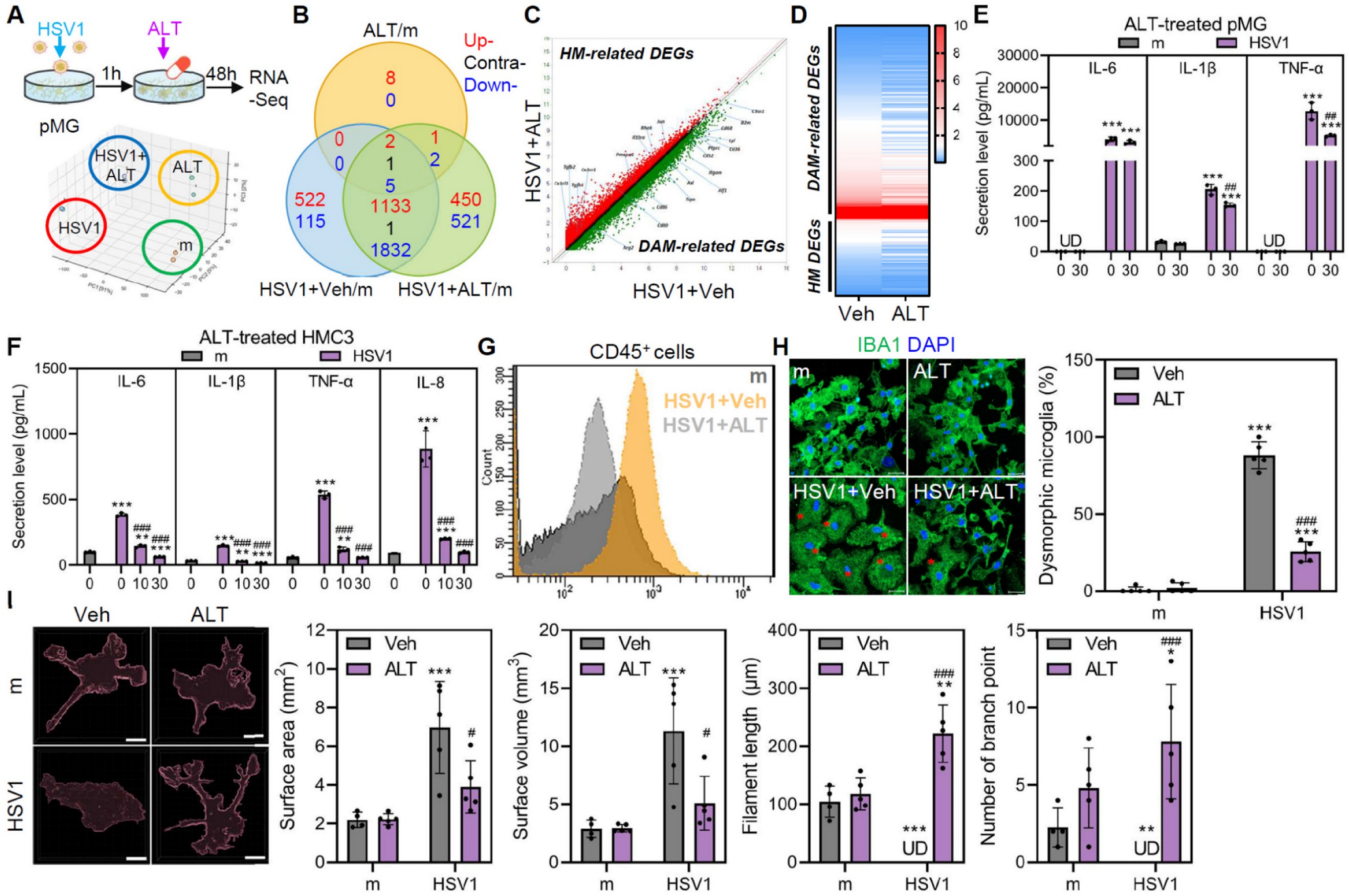
** ALT001 mitigates microglia-mediated inflammation during HSV1 infection and alters transcriptional and morphological features in HSV1-infected microglia. A** Primary microglia (pMG) were infected with mock (m) or HSV1 (MOI 10) in the presence or absence of 30 μM ALT001 (ALT) and bulk RNA sequencing was performed. A schematic overview of the experimental design is shown (upper panel) and principal component analysis was applied to score similarity of data sets (lower panel). **B** Venn diagram depicts overlapping and unique differentially expressed genes (DEGs) in HSV1-infected pMG treated with ALT. **C** A scatter plot indicates ALT treatment in HSV1-infected pMG led to alteration of mRNA expression profiles including upregulation of HM-related DEGs (red dots) and downregulation of DAM-related DEGs (green dots). **D** Heatmap showing expression profiles of microglia state-related genes** E, F** HSV1 was inoculated into pMG (MOI 10; E) or HMC3 (MOI 1; F) and cells were treated with various doses of ALT001. ELISA was performed to examine the secretion levels of IL-6, IL-8, IL-1β, and TNF-α. UD=undetermined. **G, H** pMG were infected with HSV1 (MOI 10) and treated with 30 μM ALT. At 48 hpi, cells were stained with CD45 for flow cytometry (G) and stained with IBA1 (green) for confocal microscopy (H). Dysmorphic microglia/total cells were counted using at least 50 images and the graph on the right panel shows % dysmorphic microglia. Red asterisks represent dysmorphic microglia. Scale bar=20 μm. **I** Microglia morphology was quantified using semi-automated software analysis (IMARIS). Microglia surface area, surface volume, filaments length and number of branch points were analyzed. Scale bar=10 μm. All data represent the means ± SD of at least three independent experiments. Statistical analysis: one-way ANOVA with Dunnett's post-hoc correction. *p < 0.05; **p < 0.01; ***p < 0.001, versus vehicle (Veh)-treated m-infected group. ^#^p < 0.05; ^##^p < 0.01; ^###^p < 0.001, versus Veh-treated HSV1-infected group.

Given that ALT001-mediated mitophagy mitigates mitochondrial dysfunction caused by HSV1, we postulated that ALT001 regulates microglial inflammation and homeostasis in response to HSV1 infection. Based on principal component analysis (PCA) and the Venn diagram of bulk mRNA sequencing, gene expression profile of HSV1-infected ALT001-treated groups profoundly differed from that of HSV1-infected groups (**Figure 6A**-**B**). Next, we focused on the microglial phenotypic and morphological changes as HSV1 induced DAM-associated gene signatures. As shown in **Figure 6C**, pMG treated with ALT001 after HSV1 infection showed upregulation of HM-related genes and downregulation of DAM-related genes compared to pMG infected with HSV1, suggesting that ALT001 treatment alleviated the loss of homeostatic function. Nineteen HM-related genes, including *Cx3cr1* were increased upon ALT001 treatment, whereas 112 DAM-related genes, including *Cst7*, *Fth1*, *Ccl6*, *Ctsb*, *Axl*, *Lpl*, *Lyz2*, *Trem2*, *Tyrobp,* and *B2m* were decreased compared to the HSV1-infected group (**Figure 6D**). Additionally, ALT001 attenuated IL-6, IL-1β, and TNF-α production in response to HSV1 infection in a dose-dependent manner in both human and mouse microglia (**Figure 6E-F**). These data suggest that ALT001 alleviates microglial inflammation during HSV1 infection. Furthermore, we used flow cytometry to determine the surface expression of classical DAM markers, such as CD45. Together with the modulatory effect of ALT001 on microglial gene signatures, ALT001 treatment attenuated HSV1-induced CD45 surface expression (**Figure 6G**).

Furthermore, HSV1 infection led to microglia to become rapidly swelled their soma and transformed into a dysmorphic shape; however, ALT001 significantly reduced the proportion of dysmorphic microglia, as determined by immunofluorescence labeling against IBA1 (**Figure 6H**). In addition to microglial morphology, the number of branches, filament length, surface area, and surface volume were measured using IMARIS-3D reconstruction analysis. HSV1 infection led to notable increases in surface area and volume but not in filament length or branching points (**Figure 6I**). Interestingly, ALT001 attenuated the increases in surface area and volume and losses in filaments and branches, despite these evident morphological alterations. Thus, these data indicate that ALT001 promotes morphological changes in microglia, making them less dysmorphic and reducing their phenotypic transition to DAM.

### ALT001 recovers HSV1-altered microglial phagocytosis for Aβ and prevents HSV1-induced Aβ plaque formation

**Figure 7:**
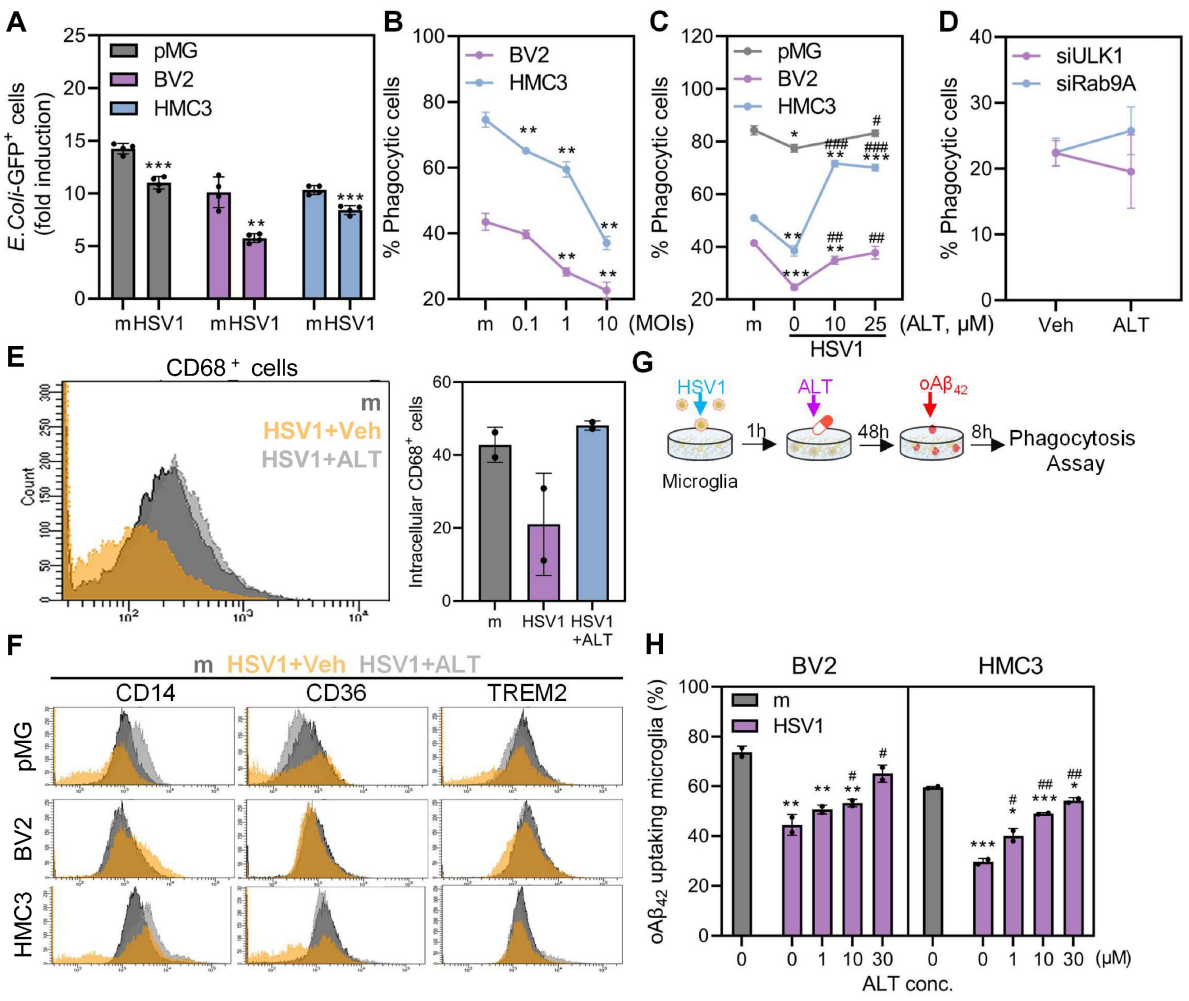
** ALT001 restores HSV1-altered microglial phagocytosis for Aβ. A** HSV1 were inoculated in primary microglia (pMG; MOI 10), BV2 (MOI 1), and HMC3 (MOI 1) for 24 or 48 h. After HSV1 infection, GFP-labeled *Escherichia coli (E. coli)* bioparticles were added to the cells and incubated for 1 h. Phagocytosed *E. coli* is presented in the graph. **B** Cells were infected with HSV1 at various MOIs and incubated with fluorescent microspheres (6 µm) for 1 h. Phagocytosis of the microspheres was quantified by flow cytometry. **C** pMG (MOI 10), BV2 (MOI 1), and HMC3 (MOI 1) were infected with HSV1 followed by treatment with various concentrations of ALT for 48 or 72 h, and cells were incubated with fluorescent microspheres (6 µm) for 1 h. Phagocytosis was quantified by flow cytometry. **D** HSV1 (MOI 1) were inoculated into HMC3 transfected with ULK1 and Rab9-specific siRNAs and cells were treated with 30 μM ALT. At 48 hpi, cells were incubated with fluorescent microspheres (6 µm) for 1h and phagocytosis was analyzed by flow cytometry. **E** Intracellular CD68 expression was analyzed by flow cytometry. **F** Surface expression of phagocytic receptors such as CD14, CD36, and TREM2 was analyzed by flow cytometry. The data are presented by representative histograms. **G** A schematic representation of microglia-mediated phagocytosis assay for oligomeric Aβ_42_ (oAβ_42_). **H** HSV1 was inoculated into BV2 and HMC3 at MOI 1 for 1 h and cells were treated with various doses of ALT. At 48 hpi, cells were treated with oAβ_42_ for 8 h and remaining oAβ_42_ was stained with Thioflavin T dye for flow cytometry analysis. All data represent the means ± SD of at least three independent experiments. Statistical analysis: one-way ANOVA with Dunnett's post-hoc correction. *p < 0.05; **p < 0.01; ***p < 0.001, versus mock (m)-infected group. ^#^p < 0.05; ^##^p < 0.01;^ ###^p < 0.01, versus HSV1-infected group.

Microglial phagocytosis serves as a crucial component of microglial defense mechanisms by effectively eliminating pathogens, neurotoxic molecules, and protein aggregates 38. Thus, we performed flow cytometry to assess the phagocytic activity of microglia during HSV1 infection. First, we observed that HSV1 infection resulted in reduced numbers of microglia that ingested fluorescein-labeled *Escherichia coli* bioparticles (**Figure 7A**). Moreover, HSV1 infection dramatically inhibited microglial phagocytosis of latex beads in a multiplicity of infection (MOI)-dependent manner in both human and mouse microglia; however, ALT001 treatment following HSV1 infection in microglia significantly recovered microglial phagocytosis of latex beads in a dose-dependent manner (**Figure 7B**-**C**).To investigate the potential role of ULK1/Rab9-mediated mitophagy by ALT001 in the recovery of microglial phagocytosis, HMC3 were transfected with Rab9 or ULK1 specific siRNA followed by HSV1 infection, and then assayed for microglial uptake of latex beads. ALT001 did not restore HSV1-altered microglial phagocytosis under Rab9 or ULK1 knockdown conditions (**Figure 7D**).

Moreover, we examined CD68 expression, which is a phagolysosomal marker in microglia, following ALT001 treatment (**Figure 7E**). Consistent with dysregulated phagocytosis, CD68 surface expression was significantly reduced in HSV1-infected microglia but was restored by ALT001 treatment. To determine the underlying mechanisms of diminished phagocytosis by HSV1, the surface expression of representative phagocytic receptors, CD14, CD36, and triggering receptor expressed on myeloid cells-2 (TREM2), was measured. As shown in **Figure 7F**, CD14 and TREM2, but not CD36, were downregulated in response to HSV1 infection, while ALT001 treatment upregulated the surface expression of phagocytic receptors, similar to mock-infected cells.

Considering that TREM2, which is involved in Aβ phagocytosis, was highly downregulated by HSV1 39, we examined the ability of Aβ clearance by microglia during HSV1 infection and ALT001 treatment. HMC3 or BV2 infected with HSV1 were exposed to ALT001 followed by oligomeric Aβ_42_ peptide treatment to measure microglial phagocytosis (**Figure 7G**). Thioflavin T staining, a fluorescent dye specific for Aβ fibrils, revealed that HSV1 infection in microglia led to insufficient removal of Aβ. On the contrary, Aβ was effectively eliminated by 30 μM ALT001-treated microglia similar to those in mock-infected cells, indicating an ability of ALT001 to enhance Aβ phagocytosis (**Figure 7H**).

To examine the effect of HSV1 infection in microglia on neurodegenerative phenotypes, we transferred conditioned medium (CM) from mock- or HSV1-infected microglia to neuronal cells. CM from HSV1-infected microglia led to a significant decrease in neuronal cell viability and reactive oxygen species (ROS) production in neurons, while cell viability and ROS production were restored in neuronal cells treated with CM from HSV1-infected microglia and ALT001 (**Figure S7**). Next, we established a microglia-neuron co-culture system to investigate whether naturally occurring Aβ fibril in HSV1-infected neurons can be removed by microglia. First, we confirmed that HSV1 infection led to Aβ accumulation and alteration in amyloidogenic pathway-related genes (*APP, BACE1,* and *PSEN1/2)* expression in neuronal cells (**Figure S8**).

Recent studies suggested the emergence of human stem cell derived brain organoids as models to capture the important aspect of HSV1 pathogenicity in CNS 40, 41. We generated cerebral organoids from hiPSCs and demonstrated that ALT001 exposure in cerebral organoids resulted in a decrease in Aβ plaque formation, as demonstrated by immunofluorescence staining with Aβ or 6E10 antibody, similar to the results from microglia-neuron co-culture model (**Figure S9A-B**). Additionally, ALT001 exposure in microglia-neuron co-cultures prevented HSV1-triggered neuronal destruction, according to fluoro-Jade C staining, which is frequently used to selectively detect degenerative neurons (**Figure S9C**) 42. In conclusion, these results suggest that ALT001 improves microglial phagocytic activities, resulting in effective elimination of HSV1-induced Aβ oligomerization and further protection against neurodegeneration.

### Exposure of ES-MG to ALT001 enhances mitophagy and phagocytosis impaired by HSV1 infection

**Figure 8:**
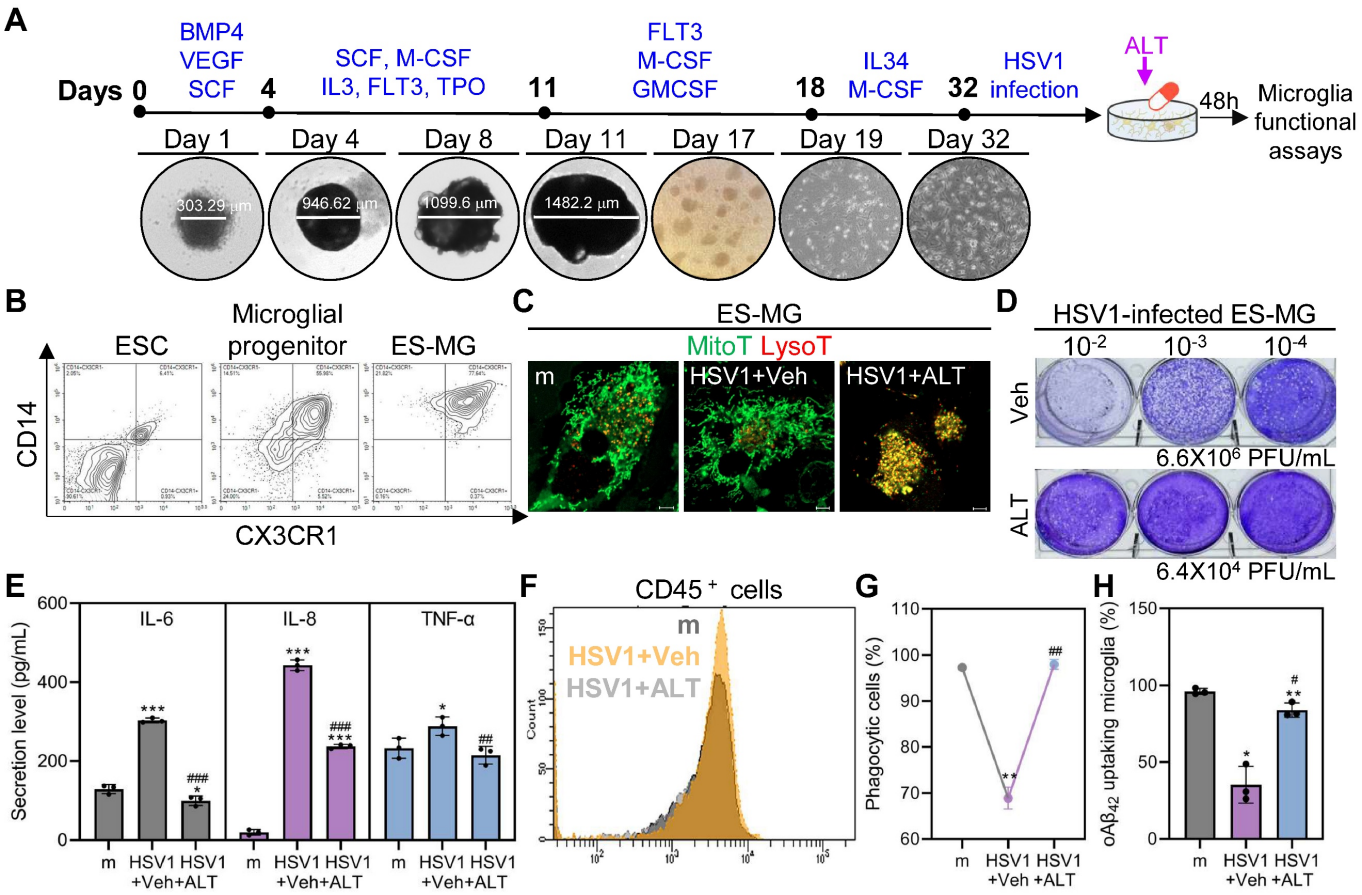
** ALT001 improves HSV1-altered microglial mitophagy and phagocytosis in human embryonic stem cell-derived microglia. A** Schematic representation of differentiation of human embryonic stem cell-derived microglia (ES-MG). **B** Embryonic stem cells (ESC), microglial progenitors (25 days post-differentiation), and ES-MG (32 days post-differentiation) were stained with antibodies against CD14 and CX3CR1 and analyzed by flow cytometry. **C** HSV1 was inoculated into ES-MG (MOI 10) and cells were treated with 30 μM ALT001 (ALT)**.** At 48 hpi, cells were stained with MitoTracker (MitoT) and LysoTracker (LysoT) to visualize mitolysosomes and analyzed by confocal microscopy. Scale bar =10 μm. **D** HSV1 was inoculated into ES-MG (MOI 10) and cells were treated with 30 μM ALT. At 48 hpi, infectious viral loads were quantified by plaque assay using supernatants. **E, F** HSV1 was inoculated into ES-MG (MOI 10) and cells were treated with 30 μM ALT. At 48 hpi, the secretion levels of IL-6, IL-8, and TNF-α were examined by ELISA (E) and cells were stained with CD45 for flow cytometry (F). **G, H** ES-MG were infected with HSV1 (MOI 10) followed by 30 μM ALT treatment for 48 h. Cells were incubated with fluorescent microspheres (6 µm) for 1 h (G) or oligomeric Aβ_42_ (oAβ_42_) for 8 h (H). Uptake of fluorescent microspheres or remaining oAβ_42_ stained with Thioflavin T was quantified by flow cytometry. All data represent the means ± SD of at least three independent experiments. Statistical analysis: one-way ANOVA with Dunnett's post-hoc correction. *p<0.05; **p<0.01; ***p < 0.001, versus vehicle (Veh)-treated mock (m)-infected group.^ #^p < 0.05; ^##^p < 0.01; ^###^p < 0.001, versus Veh-treated HSV1-infected group.

ES-MG represent an excellent model to resemble authentic microglia in terms of phenotype, surface markers, and gene expression profiles 43, 44. Embryonic stem cells (ESCs) were differentiated into microglial progenitor cells and microglial progenitor cells were successfully cultured for 14 days to mature, as shown by increased expression of CD14 and CX3CR1 (**Figure 8A-B**). Next, we tested whether mitophagy was inhibited by HSV1 by visualizing mitolysosomes. Consistent with findings in HMC3, HSV1-infected ES-MG showed reduced number of mitolysosomes, and ALT001 treatment increased mitolysosome formation in HSV1-infected cells (**Figure 8C**). We also quantitatively evaluated antiviral activities of ALT001 in HSV1 infected ES-MG. Similar to **Figure 5-6**, ALT001 exerted antiviral activities against HSV1 and downregulated HSV1-induced-pro-inflammatory cytokine secretion in ES-MG (**Figure 8D-E**). Furthermore, the surface expression of CD45 was inhibited in response to ALT001 treatment in HSV1-infected cells, suggesting that ALT001 is capable of recovering microglial phenotypic changes (**Figure 8F**). In addition to modulating innate immunity, HSV1 suppressed microglial phagocytosis, but ALT001 recovered phagocytic dysfunction in ES-MG (**Figure 8G-H**). Overall, our data demonstrate that HSV1 significantly inhibits microglial mitophagy and phagocytosis, while ALT001 improves HSV1-mediated microglial dysfunction in human ES-MG model.

## Discussion

In this study, we present a novel insight into the role of HSV1 in molecular neurodegeneration by manipulating microglial phenotype and function. Importantly, HSV1 inhibited microglial mitophagy and phagocytosis but triggered an inflammatory response, which led to neuroinflammation and insufficient Aβ degradation. Interestingly, ULK1/Rab9-dependent mitophagy activation by ALT001 triggered STING-mediated IFN antiviral responses against HSV1 to alleviate neuroinflammation and enhance Aβ phagocytosis. These findings support those from previous reports that microglial mitophagy is a pivotal determinant of neuroprotection in the prevention of AD 18.

Microglia actions are considered to play a critical role in pathological process of neurodegeneration and thus, microglial dysfunctions are one of the hallmarks of AD and other neurodegenerative diseases 5. Moreover, numerous genetic variants linked to AD are specific to microglia, emphasizing the importance of microglia in AD pathogenesis 45, 46. Other studies using single-cell or single-molecule fluorescence *in situ* hybridization sequencing have demonstrated that microglia exist in multiple states with distinct transcriptomic landscapes, including the DAM 5, 8, 47, 48. Our transcriptomic analysis revealed that HSV1-infected microglia upregulated many DAM-related genes associated with pro-inflammatory features. Moreover, HSV1-infected microglia showed severely impaired uptake of *Escherichia coli* bioparticles or Aβ, suggesting that HSV1 infection causes a phagocytic defect regardless of phagocytic cargo size or content. Notably, the induction of DAM-like state in HSV1-infected microglia led to significant suppression of phagocytic activities, although previous research indicates the localization of DAM near AD plaques 5. Given that microglial population are highly dynamic, complex and heterogeneous 5, 49, it is possible that HSV1 infection induces a morphological transformation into dysmorphic shape while exhibiting incomplete phagocytosis with increased formation of phagosomes, similar to “bubble microglia” 50-52. Furthermore, HSV1 downregulated phagocytic receptors CD14 and TREM2, which is in line with previous reports demonstrating that HSV1 inhibits TREM2 to disrupt cGAS/STING-mediated antiviral signaling 39. Our results highlight that HSV1 suppresses TREM2 expression resulting in impaired Aβ phagocytosis. It is likely that HSV1-driven TREM2 suppression reduces the number of DAM and the degree of microglial clustering around the amyloid plaques 53. Collectively, our data demonstrate that HSV1 infection alters microglial transcriptomes, morphology and function, leading to a defect in Aβ phagocytosis.

Human and mouse microglial cell lines are susceptible to HSV1 infection and offer mechanistic insights into microglial function, however, it is ideal to use primary cells derived from ESC to closely resemble authentic microglia in terms of phenotype, surface markers, and gene expression profiles. Although our results indicate similar results between primary microglia and microglial cell lines on HSV1-microglia interaction, it is interesting to note that ES-MG are highly capable of robust phagocytosis compared to that of microglial cell lines, along with very dynamic morphological changes. Therefore, ES-MG appear to be viable microglial models that are susceptible to HSV1 infection and are more similar to authentic microglia than two transformed microglial cell lines. Of note, the pathogenesis of viral encephalitis caused by HSV1 can be recapitulated in a human cerebral organoid model 40, thus, it will be intriguing to explore the effect of ALT001 in preventing HSE and its association with brain tissue damage warrants further investigation.

Multiple viruses exploit mitophagy, a selective autophagic destruction of mitochondria, to evade host immune responses and facilitate their replication 29-33. Although CCCP-mediated mitophagy was found to be inhibited by HSV1 in microglia 54, our study provides the first evidence that HSV1 directly inhibits multiple steps of PINK1/Parkin-mediated mitophagy and disturbs mitochondrial structure and function. Moreover, our study identified US3 as a virulence factor involved in the control of mitochondrial quality. Among several HSV1 autophagy inhibitory proteins, US3, originally recognized as a serine/threonine kinase, has been shown to interact with Beclin1 and induce phosphorylation of p65 and ULK1, interfering with NF-κB and autophagy pathways 23, 55. Additionally, US3 can manipulate the host cells by translocating to the mitochondria to suppress mitochondrial respiration and subvert innate immunity 23, 25, 55-57. Our data suggest a novel function of US3 as a mitophagy inhibitor by interacting with PINK1. Upon the activation, PINK1 localizes and stabilizes in mitochondrial outer membrane, resulting in the mitochondrial recruitment of Parkin for further activation of mitophagy. Therefore, we postulated that the interaction between PINK1 and US3 may affect Parkin ubiquitination as an upstream regulator of Parkin. However, further investigation is needed to elucidate how US3 interaction with PINK1 can regulate Parkin activation. Given that other HSV1 proteins, such as ICP34.5 and US11, have been demonstrated to modulate autophagy and mitophagy 21, 22, 26, we also examined the synergistic effect of mitophagy inhibition by combining US3 with US11 and our results demonstrate that US3/US11-expressing cells become highly efficient in mitophagy attenuation. Considering that neuroinflammation and mitochondrial dysfunction are representative hallmark features of AD, it will be interesting to further investigate whether there is a redundancy between US3 and other viral proteins, in HSV1-driven neurodegenerative phenotypes including Aβ and tau pathology.

Neuroinflammation is increasingly being recognized as one of the most fundamental events in the CNS by which the brain reacts to a wide range of pathogens and host-derived signals of tissue damage contributing to AD progression 58. Recurrent HSV1 infections have been proposed to be the main cause of prolonged glial cell activation and neuroinflammation in mice 59. However, little is known about the molecular mechanisms that directly connect HSV1-induced innate immunity to neuroinflammation. It is possible that HSV1 triggers cytosolic leakage of mitochondrial molecules which are known to act as damage-associated molecular patterns (DAMP) 60. The release of DAMP can be sensed by various cytosolic receptors. As an example, cGAS/STING pathway may be activated as an antiviral defense mechanism to produce type I/III IFN and may stimulate apoptosis in the CNS 34, 37. In addition to cGAS/STING pathway, NLRP3 inflammasome acts as a key DAMP-sensing receptor and is a driving force of neuroinflammation, which induces tau hyperphosphorylation and aggregation 61, 62, thus, it will be interesting to investigate the combinatory effect of an NLRP3 inhibitor with ALT001 to alleviate neuroinflammation.

Microglial mitophagy is important for Aβ and tau pathology for mitigating cognitive deficits in AD 18, 63. In addition to the previous findings that ALT001 improves cognitive impairment by mitophagy activation in neuronal cells 20, our data provide that ALT001 protects against HSV1-induced microglial dysfunction and triggers Aβ clearance by phagocytosis. A novel aspect of our study is the finding that mitophagy activation triggers cGAS/STING-mediated IFN responses to limit the release of HSV1 virions and promote microglial phagocytosis. Escoubas *et al.* demonstrated that increased IFN during developmental stress enhance microglial phagocytosis, specifically targeting neurons 53. Although IFN-responsive microglia are likely to be located near Aβ, it remains unclear whether these microglia exhibit increased phagocytic capacity for diverse cargo including Aβ. Nevertheless, our study adds novel insights to delineate underlying molecular mechanisms for the interaction between mitophagy and microglial function.

Our data highlight that ALT001-induced ULK1 activation is important for repressing HSV1 infection. The antiviral role of ULK1 has been demonstrated through the induction of IFN-stimulated genes expression 64. Although ULK1 deficiency significantly enhanced HSV1 replication, the absence of Rab9 was dispensable for the antiviral effect of ALT001. Additionally, we tested whether STING is involved in ALT001's antiviral function. As expected, STING deficient microglia exhibited increased viral titer. Interestingly, ALT001's antiviral function was abolished in STING deficient microglia, suggesting that ULK1/Rab9-mediated mitophagy exerts its antiviral functions through STING-mediated antiviral pathways (**Figure S10**). Another highlight of the antiviral effect of ALT001 is that it is more potent than the commonly used antiviral drug ACV. Considering that valacyclovir diffuses across the BBB slowly and has a penetration rate under 25% 65, it will be interesting to further evaluate immunomodulatory properties of ALT001 combined with ACV to treat HSE *in vivo*.

In summary, we identified a multifunctional role of ALT001 in modulating microglial responses and provided evidence that ALT001 can serve as a potential agent for triggering microglial mitophagy to remove dysfunctional mitochondria and inhibit neuroinflammation. ULK1/Rab9-mediated mitophagy triggered by ALT001 exerts an antiviral response to restrain HSV1 infection and promote phagocytosis against Aβ to mitigate HSV1-induced AD pathogenesis. Overall, our study highlights novel insights into the molecular mechanism of microglia activation and innate immune signaling in AD pathology upon HSV-1 infection and raises the possibility of using ALT001 as an alternative mitophagy-inducing drug for the treatment of AD and herpes infections.

## Materials and Methods

### Cells and reagents

HMC3 (CRL-3304), SH-SY5Y (CRL-2266), HEK293T (CRL-3216), and HeLa (CCL-2) were obtained from American Type Culture Collection. MN9D and BV2 were a gift from Dr. Jewook Yu (Yonsei University School of Medicine, Korea). HMC3 cells stably expressing mitochondria-targeted Keima (mt-Keima; HMC3-mt-Keima) or STING-specific shRNA (HMC3-shSTING) were generated for this study. HMC3, BV2, HEK293T, and HeLa were cultured in Dulbecco's modified Eagle medium (DMEM; CM002-050, GenDepot) supplemented with 10% fetal bovine serum (FBS; F0900-050, GenDepot) and 1% penicillin/streptomycin (P/S; 15140122, Gibco). SH-SY5Y were cultured in DMEM/F12 (11320033, Gibco) supplemented with 10% FBS and 1% P/S, whereas MN9D were incubated in cell culture dishes coated with 10 μg/ml poly-D-lysine (PDL; P0899, Sigma-Aldrich) and maintained in DMEM supplemented with 10% FBS and 1% P/S.

Carbonyl cyanide m-chlorophenyl hydrazine (CCCP; C2759) and chloroquine (CQ; C6628) were purchased from Sigma-Aldrich. Poly(I:C) (4287) and acyclovir (ACV; 2513) were purchased from Tocris. SBI-0206965 (18477) was purchased from Cayman. ALT001 has been described previously 20.

### Generation of primary microglia

#### Isolation of primary microglia from mice

All study protocols were approved by the Institutional Animal Care and Use Committee of Korea University School of Medicine, Republic of Korea. To isolate murine primary microglia (pMG), mouse pups were sacrificed, and brain tissues were collected according to a previous protocol with minor modifications 66. Purified brains were gently homogenized and filtered using a 100 μM nylon cell strainer (352360, BD Falcon). Cells were seeded in PDL-coated cell culture dishes and maintained in Ham's DMEM/F12 (11320033, Gibco) supplemented with 10% FBS, 1% P/S, 2 mM L-glutamine (25030081, Gibco), 1 mM sodium pyruvate (11360070, Gibco), and 1× MEM Non-essential amino acids (11140050, Gibco) for 14 days to obtain a confluent mixed astrocyte/microglia population. The medium was changed every 2-3 days and microglia were collected by shaking at 200 rpm for 4 h on day 14.

#### Generating microglia from human embryonic stem cells

Embryoid bodies (EBs) were generated from H1 human embryonic stem cells (ESCs) to obtain microglial progenitor and matured microglia as described previously, with minor modifications 67. Briefly, ESCs were seeded in ultra-low attachment 96-well plate (7007, Corning) with mTeSR1 medium (85850, STEMCELL) supplemented with 50 ng/ml BMP4 (795606, PeproTech), 50 ng/ml VEGF (583704, BioLegend), 20 ng/ml SCF (573902, BioLegend), and 10 µM ROCK inhibitor (1254, Tocris). On day 4, EBs with a diameter of 700-800 µm were transferred to ultra-low attachment 6-well plate containing X-VIVO 15 media (02-060F, Lonza) supplemented with 2 mM GlutaMAX (35050061, Gibco), 100 U/mL Antibiotics-Antimycotic (CA002-010, GenDepot), 0.055 mM 2-Mercaptoethanol (21985-023, Gibco), 50 ng/ml SCF, 50 ng/ml M-CSF (574804, BioLegend), 50 ng/ml IL-3 (578004, BioLegend), 50 ng/ml FLT3 (GMP-10315-HNAE1-L-AF, Sino Biological), and 5 ng/ml TPO (763702, BioLegend). On day 11, the medium was replaced with X-VIVO 15 medium supplemented with 2 mM GlutaMAX, 100 U/mL antibiotic-antimycotic solution, 0.055 mM 2-Mercaptoethanol, 50 ng/ml FLT3, 50 ng/ml M-CSF, and 25 ng/ml GM-CSF. When microglial progenitors were visible on days 18 and 25, the cells were collected and maintained in RPMI1640/GlutaMAX medium (61870036, Gibco) supplemented with 100 ng/ml IL-34 (200-34, PeproTech) and 10 ng/ml M-CSF for microglial maturation.

### Cerebral organoids generation

Cerebral organoids (CO) were generated from human induced pluripotent stem cells (hiPSCs) (Korea National Institute of Health, Korea) using STEMdiff™ Cerebral Organoid Kit (08570, STEMCELL) according to the manufacturer's recommendations. Briefly, hiPSCs were seeded into 96-well round-bottom ultra-low-attachment microplates for EB formation. After 7 days, cells were embedded in Matrigel (354227, Corning) and incubated in expansion medium for 3 days. Matrigel-embedded EBs were cultured in the maturation medium (08571, STEMCELL) for 30 days on an orbital shaker with gentle shaking, and the medium was changed every 3 days. On day 50, the maturation of CO was confirmed by immunofluorescence staining of two neuronal markers; Nestin (19483-1-AP, ProteinTech) and Pax6 (ab195045, Abcam). CO at day 50 were infected with HSV1 (100,000 PFU per CO). After 3 days, the virus-containing media were discarded, and vehicle or ALT001 was added to the culture medium. After 7 days, an immunofluorescence assay was performed.

### Cell viability and ROS assay

To measure cell viability, cells were stained with 5 μg/ml propidium iodide (PI; P4170, Sigma-Aldrich) or CCK8 (CK04-11, Dojindo). To measure apoptosis, cells were stained using a fluorescein isothiocyanate (FITC)-conjugated Annexin V Apoptosis Detection Kit (556547, BD Biosciences) according to the manufacturer's instructions and analyzed by flow cytometry. To measure ROS-producing cells, cells were stained with 20 μM 2',7'-dichlorofluorescein diacetate (DCF-DA; D399, Thermo Fisher Scientific).

### HSV1 plaque assay

HSV1 (VR-52) was obtained from Korea Bank for Pathogenic Viruses (KBPV) (Korea University School of Medicine) and propagated in Vero cells 68. For titration, Vero cells were seeded in 6-well plates with DMEM supplemented with 2% FBS and incubated overnight. Confluent Vero cells were infected with serially diluted samples and overlaid with DMEM containing 2% carboxymethylcellulose (C4888; Sigma-Aldrich). HSV1 plaques were visualized within 3 days.

### Measurement of mitophagy activity using mt-Keima

For the quantification of mitophagy, mt-Keima-expressing microglia were treated with CCCP and analyzed using a confocal microscope (LSM900, Carl Zeiss) and an LSR Fortessa X-20 flow cytometer (BD Biosciences) equipped with 405 nm/561 nm laser and BV605/PE-CF594 detector, as described previously 24. The percentage of cells undergoing mitophagy was determined by gating cells exhibiting a high ratio of emission at 561 nm/405 nm excitation.

### Bulk RNA sequencing and data analysis

Total RNA from pMG was isolated using TRIzol reagent (15596018, Invitrogen). RNA quality was assessed using Agilent 2100 Bioanalyzer with RNA 6000 Nano Chip (Agilent Technologies), and RNA quantification was performed using ND-2000 Spectrophotometer (Thermo Fisher Scientific). RNA was subjected to bulk RNA sequencing using Illumina NovaSeq 6000 platform (Illumina). Quality control of the raw sequencing data was performed using Fast QC software. Adapter and low-quality reads were removed using FastQ 69. The trimmed reads were mapped to the mm10 with GENCODE version M21 genome using a STAR aligner 70. Reads were quantified using Salmon 71 and processed using TMM + CPM normalization method with EdgeR 72. Data mining and graphic visualization were performed using ExDEGA (Ebiogen). Genes with log_2_FC < 1 and p < 0.05 were identified as differentially expressed genes (DEGs). Heatmap, principal component analysis (PCA) scatter plots, and Venn diagrams were generated based on the DEGs. Significant DEGs were subjected to functional annotation analysis using DAVID Bioinformatics Resources 6.8 (https://david.ncifcrf.gov/summary.jsp), and Kyoto Encyclopedia of Genes and Genomes (KEGG) pathway enrichment analysis was performed using the Database for Annotation, Visualization, and Integrated Discovery v6.8. Bulk RNA-sequencing data has been deposited to NCBI GEO database under the accession number: GSE278015.

### Plasmids and siRNA or shRNA-mediated gene knockdown

Generation of LC3-GFP, Parkin-GFP, mt-dsRED, PINK1-V5, Parkin-MYC, Rab9-YFP, pLV-mt-Keima, pLV-shRab9, and pLV-shULK1 have been described previously 20, 29, 31. HSV1 US3 and US11 were synthesized and confirmed by sequencing, followed by cloning into pBHA vector (Bioneer). pLV-US3-GFP was generated by subcloning the synthesized pBHA-US3 into a pLV-eGFP (Addgene) vector. The cells were transiently transfected with DNA plasmids using Lipofectamine 2000 (11668027, Invitrogen) or with control scrambled, Rab9, ULK1, STING-specific siRNAs (Bioneer) using Lipofectamine RNAiMAX (13778100, Invitrogen) according to the manufacturer's instructions.

### Luciferase assay

Cells were transfected with pIP-10-Luc or pNF-κB-Luc or pRL-TK, and genes of interest were co-transfected. poly(I:C) was transfected into cells for stimulation for 6 h at 24 h after DNA transfection. Supernatants were collected and measured for luciferase activities using Dual-Glo Luciferase Assay Kit (E2920, Promega).

### Transmission electron microscopy (TEM)

Samples were prepared as previously described 31. Briefly, HSV1-infected pMG and HMC3 cells were pre-fixed with 2.5% glutaraldehyde for 4 h followed by post-fixation with reduced osmium tetroxide for 1.5 h at 4 °C. After washing thrice, the cells were dehydrated using a graded series of ethanol and embedded in Epon resin. Ultrathin sections were prepared and stained with uranyl acetate and lead nitrate. Images were captured using a Hitachi H-7000 electron microscope.

### Immunofluorescence

Cells were fixed with 4% paraformaldehyde, permeabilized with 0.1% Triton X-100 diluted in PBS, and blocked with 2.5% bovine serum albumin (BSA) for 20 min at each step. Primary antibodies against HSV1 ICP0 (SC-53070, Santa Cruz), 6E10 (803014, BioLegend), or Aβ (36-6900, Invitrogen) were prepared in 0.5% BSA in PBS and incubated with cells for 1 h, followed by secondary antibody incubation using anti-mouse Alexa594 (A11005, Invitrogen), and anti-rabbit Alexa 488 (A11008, Invitrogen). For nuclear staining, 1 μg/ml 4',6-diamidino-2-phenylindole (DAPI; D9542, Sigma-Aldrich) were incubated with cells for 10 min. Stained cells were imaged using a confocal microscope and analyzed using a Zen desk (Carl Zeiss).

To visualize mitolysosomes, the cells were treated with MitoTracker and LysoTracker for 30 min and analyzed using a confocal microscope (LSM900, Carl Zeiss). To quantify the degenerating neurons, cells were stained with Fluoro-Jade C (FJC; TR-100-FJT, Biosensis) according to the manufacturer's recommendations. FJC-positive cells were counted from at least 300 cells for each condition, compiled from at least three experiments, and calculated as % FJC-positive cells/total DAPI-positive cells.

### Microglial morphometric analysis

Cells were fixed with 4% paraformaldehyde, permeabilized with 0.1% Triton X-100 diluted in PBS, and blocked with 2.5% bovine serum albumin (BSA) for 20 min at each step. Primary antibodies against IBA1 (019-19741, FUJIFILM Wako) were prepared in 0.5% BSA in PBS and incubated with cells for 1 h, followed by secondary antibody incubation using anti-rabbit Alexa 488 (A11008, Invitrogen). For nuclear staining, 1 μg/ml 4',6-diamidino-2-phenylindole (DAPI; D9542, Sigma-Aldrich) were incubated with cells for 10 min. Stained cells were imaged using a confocal microscope and analyzed using a Zen desk (Carl Zeiss) and IMARIS 10.1.0 3D analysis software (Oxford Instruments) where the semi-automated analysis was performed. Morphometric analysis was performed on IBA1-positive microglia whose processes were entirely within 3D Z-stack. The surface feature was used to quantify cell sphericity, and automated filament tracing was used to quantify the length of the process and the number of branch points.

### Quantification of microglial phagocytosis

To determine microglial phagocytic activities, *Escherichia coli* (K-12 strain) BioParticles™ (V6694, Invitrogen) or fluoresbrite® BB Carboxylate Microspheres (6.00 µm) (19102-2, Polysciences) were pre-opsonized, added to the cells and incubated for 1 h. The cells were washed twice to remove non-engulfed beads and excited with a UV laser (355 nm) with emission detected at 450 ± 50 nm by DAPI detector using BD LSR Fortessa X-20 flow cytometer (BD Biosciences). For Aβ-specific phagocytosis assay, oligomeric Aβ-treated cells were stained with Thioflavin T (T3516, Sigma-Aldrich).

To measure surface protein expression, the microglia were harvested, stained with anti-CD45 (563890, 563880, BD Biosciences), anti-CD11b (12-0112-82, Thermo Fisher Scientific), anti-CD14 (123307, 325603, BioLegend), anti-CX3CR1 (341611, BioLegend) and anti-CD36 (336203, 102607, BioLegend) antibodies for 1 h, and analyzed by flow cytometry. Cells were stained with anti-TREM2 (MAB-17291, R&D Systems), followed by secondary antibody incubation with anti-rat Alexa 488 (A-11006, Invitrogen). To measure intracellular CD68 positive phagolysosomes, cells were fixed with BD Cytofix/Cytoperm fixation/permeabilization kit (554714, BD Biosciences) followed by staining with anti-CD68 (333809, BioLegend).

### Immunoblotting analysis

Samples were subjected to 8-15% SDS-PAGE and transferred onto polyvinylidene difluoride (PVDF; IPVH00010, Millipore) membranes. After blocking with 5% skim milk in Tris-buffered saline (TBS; T2008, Biosesang) supplemented with 0.1% Tween-20 (TBS/Tw) for 1 h, membranes were incubated with primary antibodies at 4 °C overnight. The following antibodies were used: HSV1 ICP0 (SC-53070, Santa Cruz), HSV1/2 gB (SC-56987, Santa Cruz), HSV1/2 gD (SC-69802, Santa Cruz), p62 (8025, Cell Signaling Tech), LC3 (3868, Cell Signaling Tech), Actin (AM1021B, Abcepta), Tubulin (663751-2-AP, ProteinTech), phospho-Ubiquitination (Ser56) (62802, Cell Signaling Tech), Parkin (4211, Cell Signaling Tech), phospho-PINK1 (46421, Cell Signaling Tech), PINK1 (23274-1-AP, ProteinTech), COX II (ab198286, Abcam), GFP (SC-9996, Santa Cruz), MYC (16286-1-AP, ProteinTech), TOM20 (SC11415, Santa Cruz), TOM70 (14528-1-AP, ProteinTech), GM130 (610822, BD Bioscience), Calnexin (10427-2-AP, ProteinTech), phospho-ULK1 (Ser555) (5869, Cell Signaling Tech), ULK1 (8054, Cell Signaling Tech), phospho-STING (72971, Cell Signaling Tech), STING (13647, Cell Signaling Tech), phospho-TBK1 (5483, Cell Signaling Tech), TBK1 (5483, Cell Signaling Tech), phospho-IRF3 (29047, Cell Signaling Tech), IRF3 (SC-33641, Santa Cruz), Rab9 (5118, Cell Signaling Tech), PARP (9542, Cell Signaling Tech), Caspase 7 (12827, Cell Signaling Tech), Caspase 9 (9508, Cell Signaling Tech), and Caspase 3 (14220, Cell Signaling Tech). After incubation in primary antibody, membranes were washed and incubated with secondary HRP-conjugated anti-mouse or rabbit antibodies, followed by imaging using the Fusion Solo Imaging System (Vilber Lourmat STE). Quantification of immunoblots was performed by ImageJ (National Institutes of Health).

### Co-immunoprecipitation

HEK293T seeded in 60 mm dishes were transfected with the indicated plasmids, incubated for 24 h, and lysed in immunoprecipitation lysis buffer (87787, Thermo Fisher Scientific) supplemented with a complete protease inhibitor cocktail (1183617001, Roche). DynaBeads protein G (10003D, Thermo Fisher Scientific) was incubated with 1 μg primary antibodies against MYC (16286-1-AP, ProteinTech) or PINK1 (23274-1-AP, ProteinTech) for 1 h for conjugation. After removing cell debris via centrifugation, the antibody-conjugated beads were incubated with 500 μg cell lysates overnight at 4 °C, with rotation. Immunoprecipitates were eluted in 2× Laemmli sample buffer or SDS sample buffer and analyzed by immunoblotting to detect interactions between proteins.

### Quantitative RT-PCR

Total RNA was extracted using TRIzol reagent (15596018, Invitrogen) or DirectZol RNA kit (R2072, Zymo Research). cDNA was reverse-transcribed using High-Capacity cDNA Reverse Transcription Kit (4368814, Thermo Fisher Scientific). Quantitative PCR was performed in triplicate using Power SYBR Green Master Mix (4368577, Invitrogen). Primer sequences have been previously reported 30, 31. To measure total mitochondrial DNA, genomic DNA was extracted from cells using phenol-chloroform and ethanol method as previously described 73. Relative mitochondrial DNA level was determined based on the 2^-ΔΔCT^ method normalized against 18s rRNA.

### Measurement of mitochondrial metabolic flux

Cells were seeded onto mini-plates (103022-100, Agilent) and incubated in a non-CO_2_ incubator for 1 h before the oxygen consumption rate (OCR) was measured. Seahorse XFp Cell Mito Stress Test Kit (103010-100, Agilent) was used according to the manufacturer's instructions. Briefly, after measuring basal OCR, 1 μM oligomycin, 2 μM fluoro-carbonyl-cyanide phenylhydrazone (FCCP) or 1 μM rotenone/antimycin A (Rot/AA) were added to the cells at the indicated time points.

### Cytokine and chemokine secretion measurement

Secreted levels of cytokines and chemokines, including IL-6 (DY206, DY406), IL-8 (DY208), IL-1β (DY201, DY401), TNF-α (DY210, DY410), and IFN-β (DY814), were measured using conditioned media from cells by ELISA kit (R&D Systems) or a magnetic Luminex screening assay with a Human Premixed Multi-Analyte Kit (R&D Systems).

### Statistical analysis

Data are presented as the mean ± standard deviation from at least three independent experiments. Data were analyzed using Student's t test or one-way ANOVA with Dunnett's post-hoc correction. All statistical analyses were performed using GraphPad Prism 9.0 software.

## Supplementary Material

Supplementary figures.
