# Supplementary material for: Pharmacological targeting of mitophagy via ALT001 improves herpes simplex virus 1 (HSV1)-mediated microglial inflammation and promotes amyloid β phagocytosis by restricting HSV1 infection
Source: Theranostics. 2025 Mar 31;15(11):4890–908. doi: 10.7150/thno.105953 (PMC12036882; doi:10.7150/thno.105953)
Supplement: Supplementary file 1 — Supplementary figures. [file thnov15p4890s1.pdf]

Supplementary materials

Pharmacological targeting of mitophagy via ALT001 improves herpes simplex virus 1 (HSV1)-mediated microglial inflammation and promotes amyloid β phagocytosis by restricting HSV1 infection

Soo-Jin Oh[1], Young Yeon Kim[2], Ruiying Ma[1, 3], Seok Tae Choi[4], Se Myeong Choi[5], Jong Hyun Cho[5], Ji-Yeun Hur[6], Yongjin Yoo[1, 3], Kihoon Han[1, 3], Hosun Park[4], Jeanho Yun[2]*, Ok Sarah Shin[1,7]*

[1] BK21 Graduate Program, Department of Biomedical Sciences, College of Medicine, Seoul, Republic of Korea
[2] Department of Translational Biomedical Sciences, Graduate School of Dong-A University, Busan, Republic of Korea
[3] Department of Neuroscience, Korea University College of Medicine, Seoul, Republic of Korea
[4] Department of Microbiology, College of Medicine, Yeungnam University, Daegu, Republic of Korea
[5] Department of Medicinal Biotechnology, College of Health Sciences, Dong-A University, Busan, Republic of Korea
[6] Department of Biological Sciences, Ajou University, Suwon, Republic of Korea
[7] Department of Convergence medicine, College of Medicine, Korea University Guro Hospital, Seoul, Republic of Korea

* Co-correspondence: oshin@korea.ac.kr (OSS); Tel.: 82-2-2626-3280, yunj@dau.ac.kr (JY); Tel.:82-51-240-2919

Figure S1.

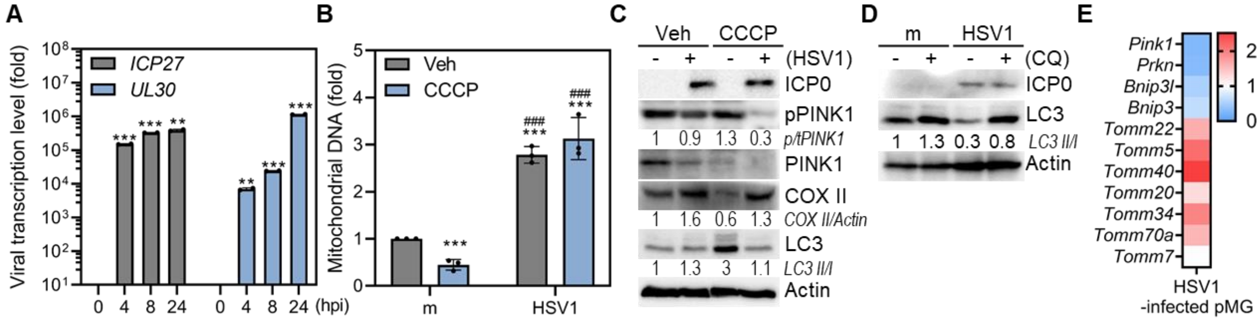

Fig. S1. Suppression of mitophagy by HSV1 in murine microglia

A Murine microglial cell line **(**BV2) were infected with HSV1 (MOI 1). At indicated time points, RNA was isolated from cells, and *HSV1 ICP27* and *UL30* expression levels were analyzed by qRT-PCR. **B, C** BV2 were infected with HSV1 at MOI 1 for 24 h, followed by vehicle control (Veh) or 20 μM CCCP treatment for 2 h. Mitochondrial DNA copies were quantified by PCR (B) and an immunoblot was performed to determine protein expression of HSV1 ICP0, phospho-PINK1 (p-PINK1), PINK1, Mitochondria-encoded cytochrome C oxidase II (COX II), LC3, and Actin (C). Band quantification relative to Actin is shown below the blot**. D** BV2 were infected with HSV1 (MOI 1) for 24 h, followed by 10 μM chloroquine (CQ) treatment for 8 h. Immunoblot analysis was performed to examine the levels of HSV1 ICP0, LC3, and Actin. **E** A heatmap was generated using gene expression profiles from bulk RNA-sequencing of HSV1-infected pMG compared with mock-infected pMG (MOI 10). Data are presented as means ± SD (n =3 biological replicates; *p* values were calculated by Student's t test). ** $p < 0.01$; ***$p < 0.001$, *compared with mock (m)-infected Veh-treated group.* ###$p < 0.001$, *compared with mock-infected CCCP-treated group.*

Figure S2.

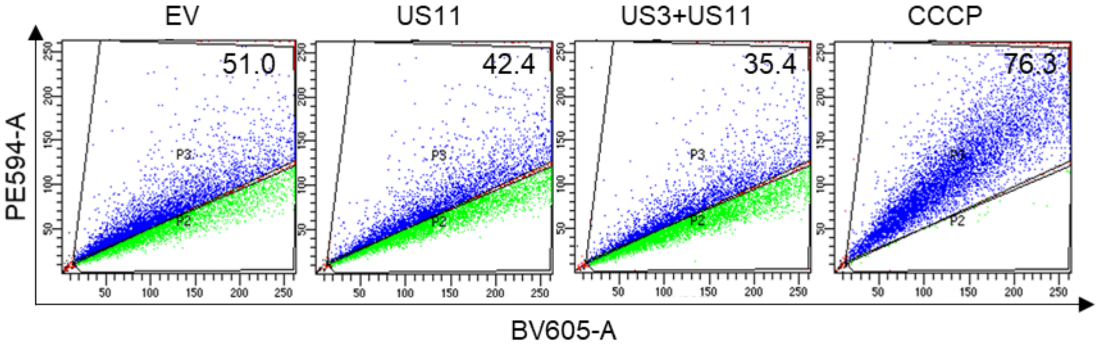

Fig. S2. Suppression of microglial mitophagy by HSV1-encoded US11

HMC3-mt-Keima were transfected with either empty vector (EV), US3, US11, or US3+US11-encoding plasmids and mitophagy activity were assessed by flow cytometry. Cells treated with 20 μM CCCP for 2 h were used as positive control for mitophagy induction, as detected by mt-Keima.

Figure S3.

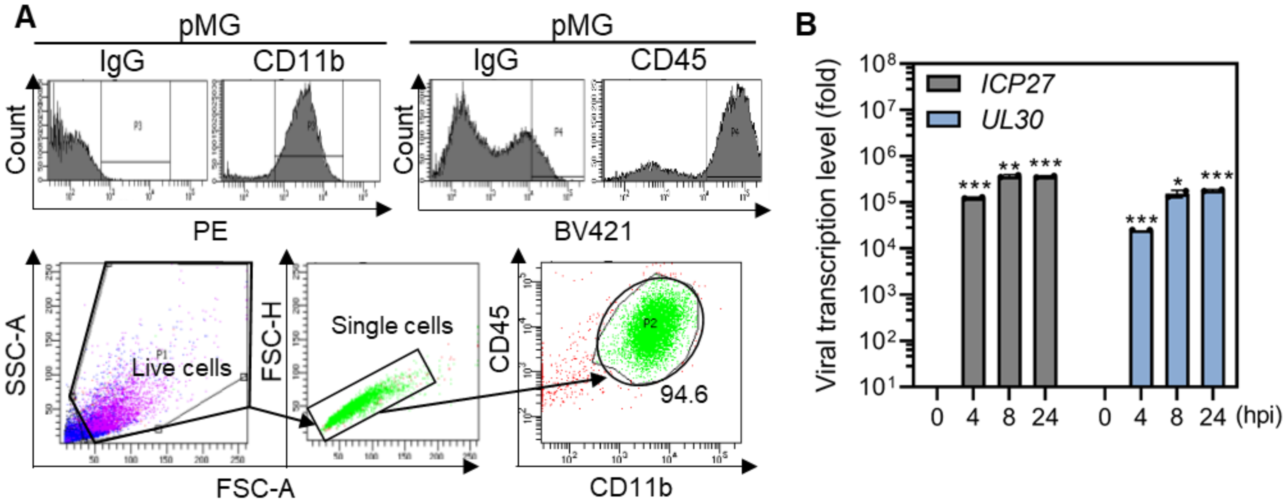

Fig. S3. Characterization of isolated primary murine microglia (pMG)

A Mixed glial cells (MxG) were isolated from mouse pups and grown for 14 days. After 14 days, primary murine microglia were isolated from MxG and stained with anti-CD11b and anti-CD45 antibodies for flow cytometry. **B** pMG were infected with HSV1 at MOI 10. At indicated time points, RNA was isolated from cells, and qRT-PCR was performed to examine the level of *HSV1 ICP27* and *UL30*. Data are presented as means ± SD (n =3 biological replicates; *p* values were calculated by Student's t test). *** $p < 0.01$; ***$p < 0.001$, versus mock (m)-infected group.*

68 **Figure S4.**

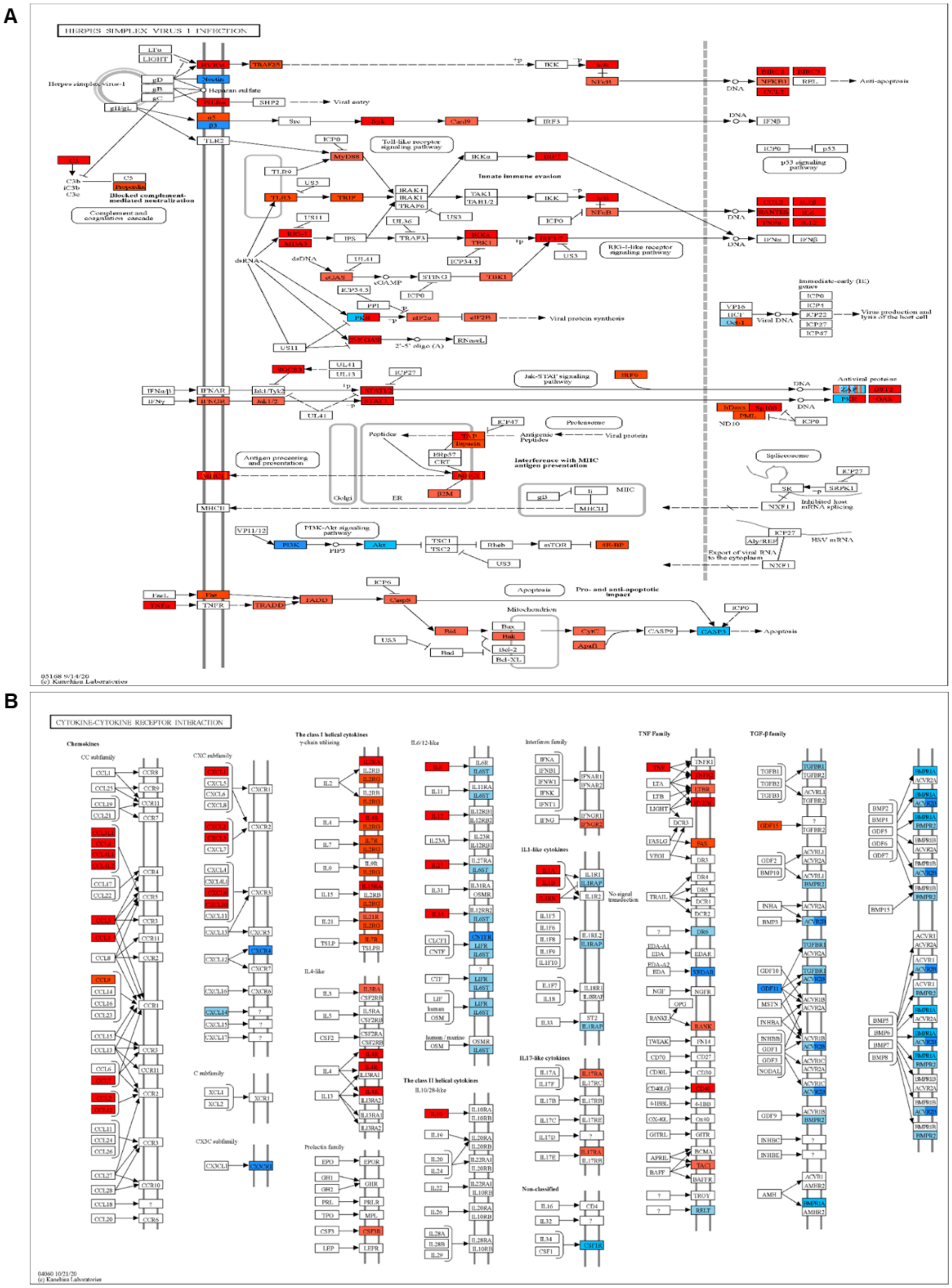

69

Fig. S4. Alterations in HSV-1-associated host antiviral signaling pathway in microglia

A, B KEGG pathway map show upregulated (red) and downregulated (blue) genes in HSV1-infected pMG (MOI 10) compared with mock-infected pMG. Herpes simplex virus 1 infection (KEGG #; mmu05168) (A) and Cytokine-Cytokine receptor interaction (KEGG #; 04060) (B) were generated.

Figure S5.

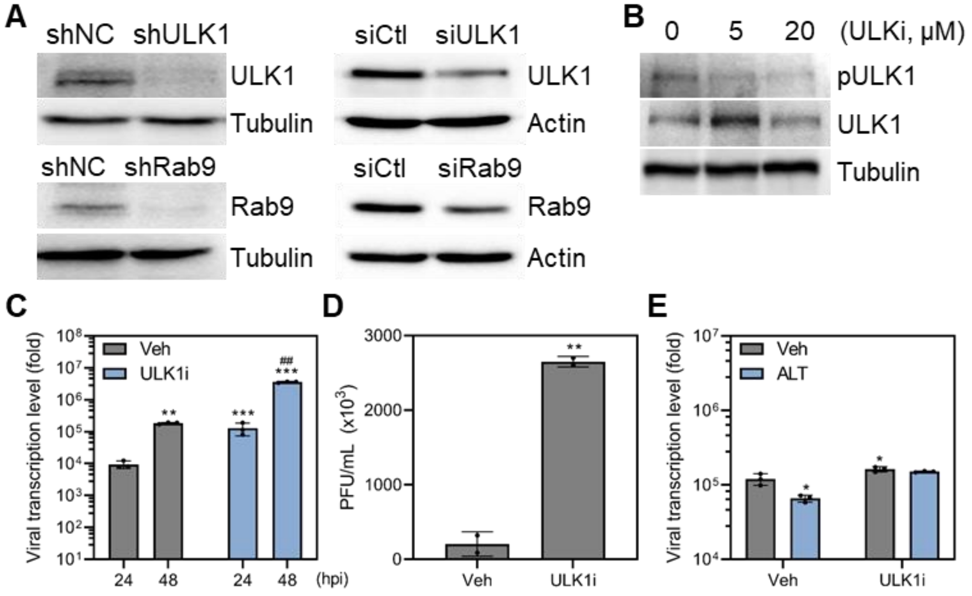

Fig. S5. ULK1 modulates HSV1 replication in microglia

A HMC3 were transfected with scrambled negative control (shNC), ULK1-specific shRNA (shULK1), and Rab9-specific shRNA (shRab9) or control siRNA (siCtl), ULK1-specific siRNA (siULK1), and Rab9-specific siRNA (siRab9). The knockdown efficiency of shRNA or siRNA was validated by immunoblot analysis showing protein levels of ULK1, Rab9, Actin, and Tubulin. **B** HMC3 were treated with 20 µM ULK1 inhibitor (SBI-0206965, ULK1i) for 48 h and a representative immunoblot analysis of phospho-ULK1 (p-ULK1), ULK1, and Tubulin is shown. **C, D** HSV1 was inoculated in HMC3 (MOI 1) and cells were treated with 20 µM ULK1i. *HSV1 ICP27* mRNA levels were analyzed by qRT-PCR (C) and a plaque assay were performed to determine HSV1 titer (D). *$^*p < 0.05$; $^{**}p < 0.01$; $^{***}p < 0.001$, versus vehicle (Veh)-treated group at 24hpi. $^{\#\#}p < 0.01$, versus Veh-treated at 48hpi.* **E** HSV1 was inoculated in HMC3 at MOI 1 and cells were treated with 30 µM ALT001 (ALT) and 20 µM ULK1i. At 48 hpi, RNA was isolated from cells, and *HSV1 ICP27* mRNA levels were analyzed by qRT-PCR. *$^*p < 0.05$, versus Veh-treated group*. Data are presented as means ± SD (n =3 biological replicates; *p* values were calculated by Student's t test).

Figure S6.

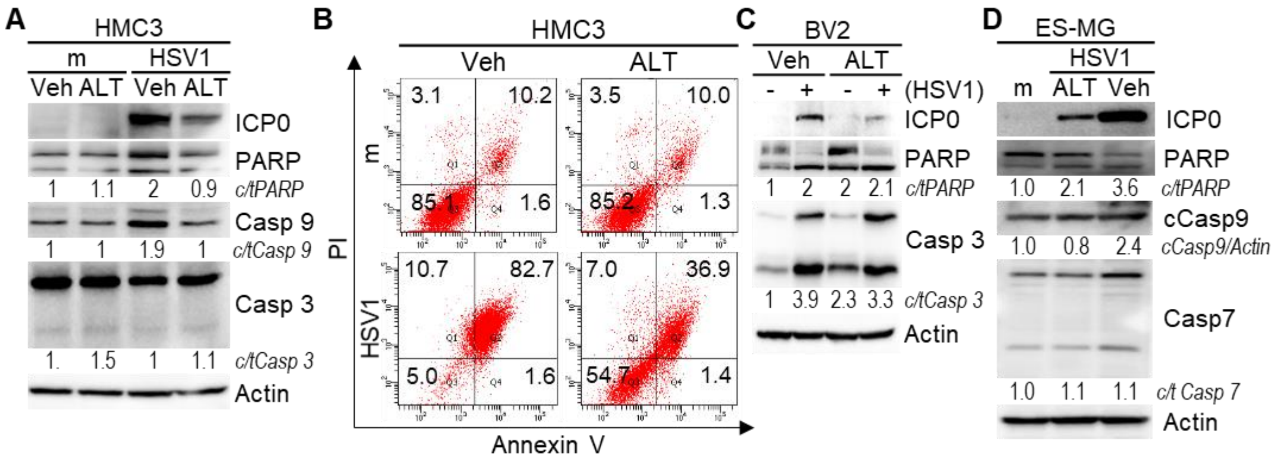

Fig. S6. ALT001 suppresses HSV1-induced apoptosis in microglia

A, B HSV1 were inoculated in HMC3 (MOI 1) and cells were treated with vehicle (Veh) or 30 μM ALT001 (ALT). At 48 hpi, HSV1 ICP0, cleaved (c) PARP/total (t) PARP, cleaved caspase 9/total caspase 9 and cleaved caspase 3/total caspase 3 expression is shown by immunoblot analysis (A). Actin was used as a protein loading control. Numbers below the blot represent quantified band intensity by densitometric analysis. Cells were stained with Annexin V-FITC/PI and apoptosis was analyzed by flow cytometry (B). **C ,D** BV2 (C) or ES-MG (D) cells were infected with HSV1 (MOI 1) in the presence or absence of 30 μM ALT. HSV1 ICP0, cleaved PARP/total PARP, and cleaved caspase 3/total caspase 3 is shown by immunoblot analysis. Images are representatives of three independent experiments. Band quantification relative to Actin is shown below the blot**.**

Figure S7.

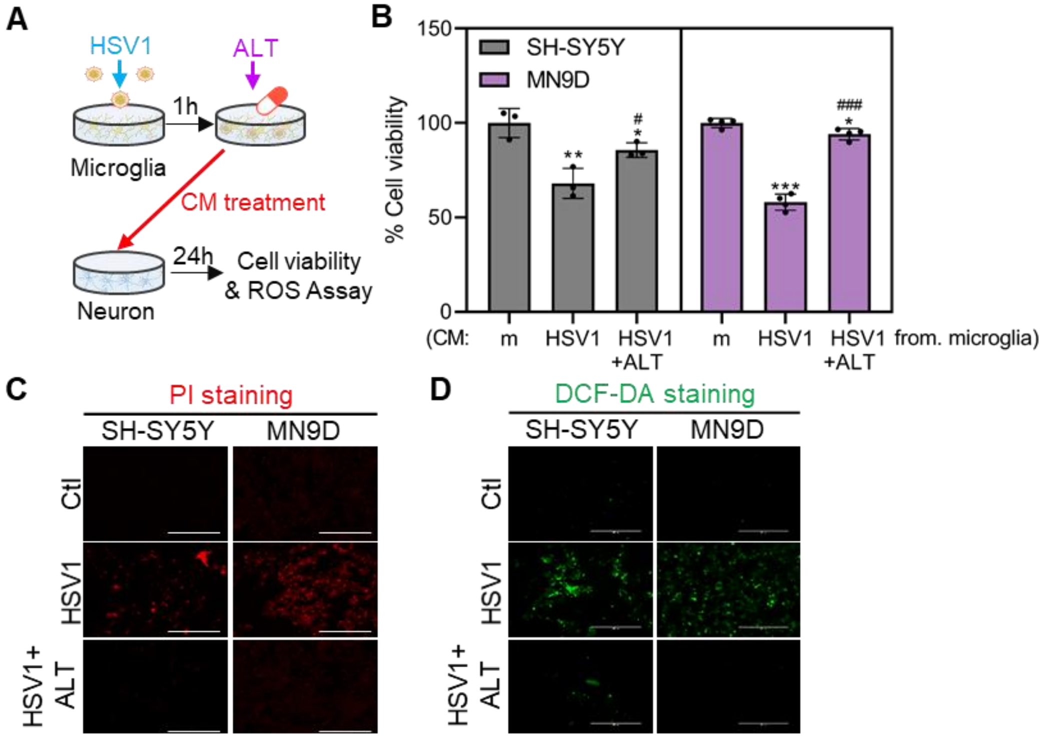

Fig. S7. ALT001 suppresses HSV1-mediated cell death in neuronal cells

A A schematic representation of conditioned media (CM) treatment from microglia to neuronal cells is shown. **B** Human and mouse neuronal cells (SH-SY5Y and MN9D) were treated with CM collected from mock (m)-, HSV1-, or HSV1-infected with ALT001 (HSV1+ALT) microglia. After 24 h, CCK8 assay was performed to measure cell viability. Statistical analysis: one-way ANOVA with Dunnett's post-hoc correction. *$*p < 0.05$; $**p < 0.01$; $***p < 0.001$, versus CM from m-infected group.* $^{\#}p < 0.05$, $^{\#\#\#}p < 0.001$, *versus CM from Veh-treated HSV1-infected group.* **C, D** CM-treated neuronal cells were stained with propidium iodide (PI) (red) or 2',7'-Dichlorofluorescein Diacetate (DCF-DA) (green). Stained cells were analyzed by EVOS FL Auto 2 Imaging system. Scale bar = 200 µm.

Figure S8.

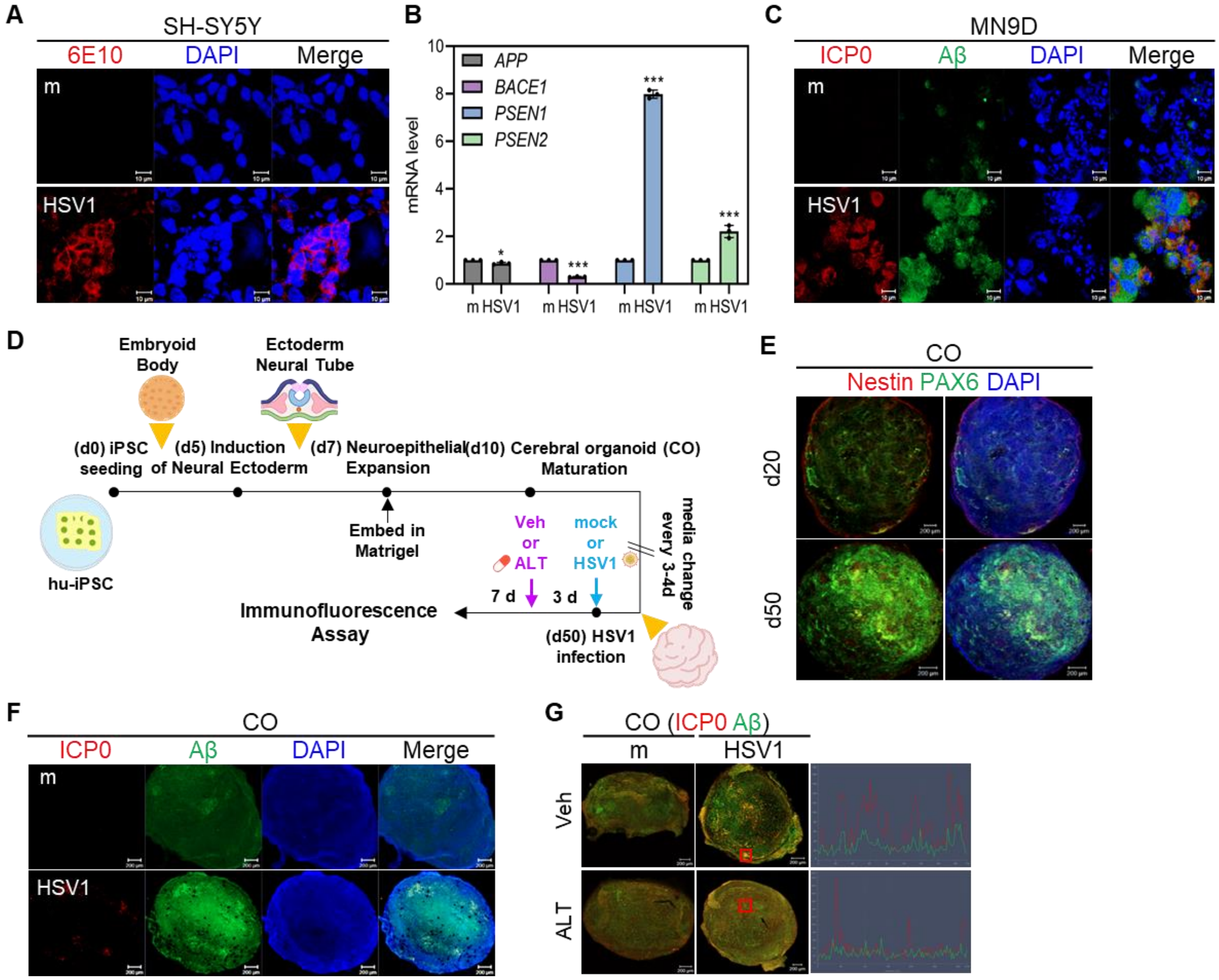

Fig. S8. HSV1 triggers Aβ plaque formation in neuron and cerebral organoids (CO)

A, B Human neuroblastoma cells (SH-SY5Y) were infected with HSV1 (MOI 1) for 48 h and stained against 6E10 (red) antibodies for confocal microscopy analysis (A) and mRNA levels of *amyloid beta precursor protein* (*APP*), *beta-secretase 1* (*BACE1*), *presenilin 1* (*PSEN1*), and *presenilin 2* (*PSEN2*) was measured by qRT-PCR (means ± SD; n = 3) (B). Statistical analysis; Student's t test. **p < 0.05; ***p < 0.001, versus mock (m)-infected group.* Scale bar=10 μm. **C**

Mouse dopaminergic neuronal cells (MN9D) were infected with HSV1 (MOI 1) for 48 h and were stained with HSV1 ICP0 (red) and Aβ (green) for confocal microscopy analysis. Scale bar =10 µm. **D** Schematic representation of HSV1 infection in cerebral organoid (CO) model is shown. **E** On day 20 or 50 after CO generation, CO were stained with neuronal marker, Nestin (red) and PAX6 (green). Scale bar=200 µm. **F** On day 50 of CO generation, CO were infected with HSV1 (10^5 PFU) for 3 days followed by immunofluorescence staining against HSV1 ICP0 (red) and Aβ (green) for confocal microscopy analysis. Scale bar =200µm. **G** CO were infected with HSV1 (10^5 PFU) for 3 days and incubated with vehicle (Veh) or 30 µM ALT001 (ALT) for 7 days. CO were stained with HSV1 ICP0 (red) or Aβ (green) for confocal microscopy analysis. The graph on the right represents the quantification of fluorescence intensity in the red box area of images. Scale bar =200 µm.

Figure S9.

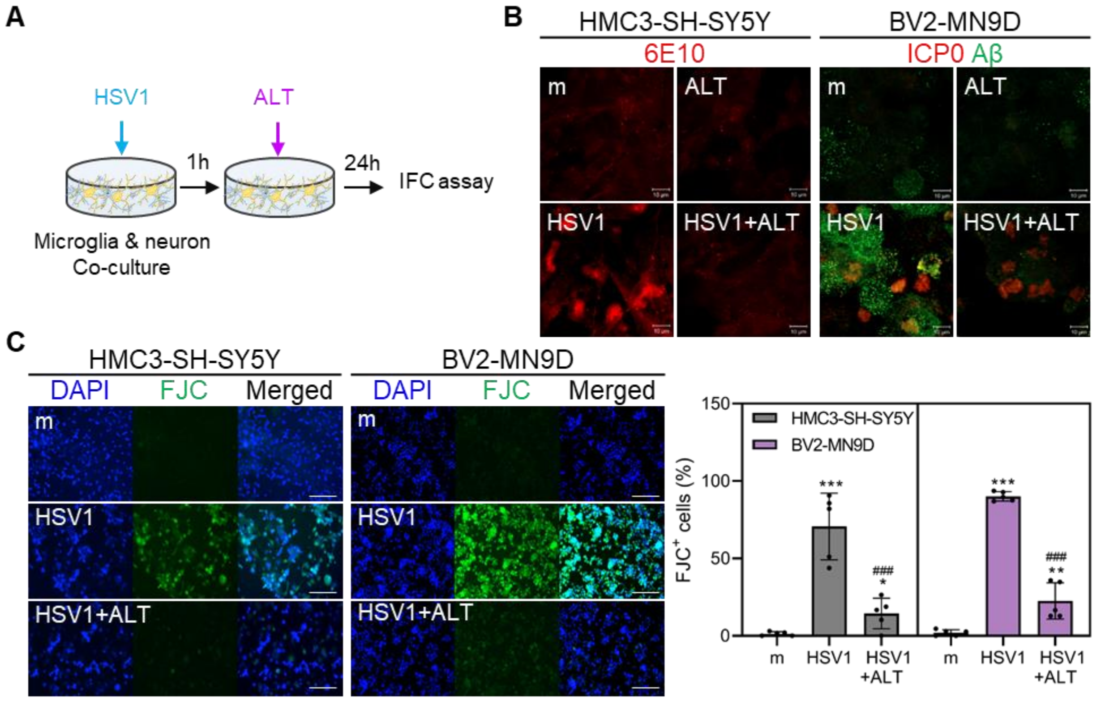

Fig. S9. ALT001 reduces degeneration of neurons in microglia-neuron co-culture model

A A schematic representation of microglia & neuron co-culture system is shown. HSV1 was inoculated in human microglia-neuron (HMC3-SH-SY5Y) or murine microglia-neuron (BV2-MN9D) co-culture model and these cells were treated with 30 μM ALT001 (ALT). **B** At 24 hpi, cells were stained with 6E10 (red), Aβ (green) or HSV1 ICP0 (red) for confocal microscopy analysis. Scale bar =10 μm. **C** cells were stained with FJC (green). % FJC-positive cells were calculated using at least 5 different images. Scale bar=200 μm. Data are expressed as the means ± SD of at least three independent experiments. Statistical analysis: one-way ANOVA with Dunnett's post-hoc correction. **$p<0.05$; **$p<0.01$; ***$p < 0.001$, versus vehicle (Veh)-treated mock (m)-infected group. $^{\#}p < 0.05$; $^{\#\#\#}p < 0.001$, versus Veh-treated HSV1-infected group.*

Figure S10.

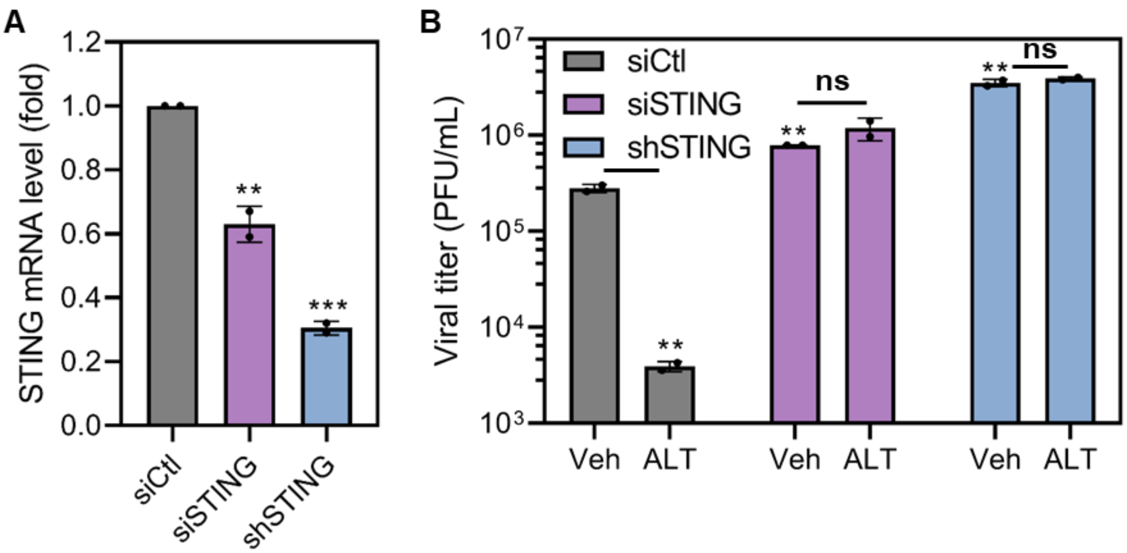

Fig. S10. ALT001-mediated mitophagy exerts antiviral response against HSV1 via STING

A STING mRNA expression level was measured in control siRNA-transfected HMC3 (siCtl), STING-specific siRNA-transfected HMC3 (siSTING), and shSTING-stably expressing HMC3 (shSTING) by qRT-PCR. **B** STING knockdown cells by siRNA or shRNA introduction were infected with HSV1 at MOI 1 in the presence or absence of vehicle (Veh) or 30 μM ALT001 (ALT). Supernatant was collected at 48 hpi and a plaque assay was performed in Vero cells using the supernatant. Statistical analysis: one-way ANOVA with Dunnett's post-hoc correction. ***p<0.01; ***p < 0.001, versus siCtl-transfected Veh group*. ns=non-significant.
